# Highly Efficient and Stable Binary Cross‐Linkable/ Self‐Assembled Organic Nonlinear Optical Molecular Glasses

**DOI:** 10.1002/advs.202304229

**Published:** 2023-09-10

**Authors:** Lian Zhang, Fenggang Liu, Ruoxi Yang, Fuyang Huo, Weijun Zhang, Yu Zhang, Chuying Liu, Chunngai Hui, Jiahai Wang

**Affiliations:** ^1^ School of Chemistry and Chemical Engineering Guangzhou University Guangzhou 510006 P. R. China; ^2^ Huawei Technologies Bantian Industrial Base Shenzhen 518129 P. R. China

**Keywords:** alignment stability, chromophores, cross‐linking, nonlinear optics, self‐assembly

## Abstract

The development of electro‐optical materials with high chromophore loading levels that possess ultrahigh electro‐optic coefficients and high long term alignment stability is a challenging topic. Anthracene‐maleimide Diels–Alder (DA) reaction and π–π interaction of Anthracene‐pentafluorobenzene and benzene‐pentafluorobenzene are developed for making highly efficient binary cross‐linkable/self‐assembled dendritic chromophores FZL1‐FZL4. A covalently or non‐covalently cross‐linked network is formed by DA reaction or π–π interaction after electric field poling orientation, which greatly improves the long‐term alignment stability of the materials. An electro‐optic coefficient up to 266 pm V^−1^ and glass transition temperature as high as 178 °C are achieved in cross‐linked film FZL1/FZL2, and 272–308 pm V^−1^ is achieved for self‐assembled films FZL1/FZL4 and FZL3/FZL4 due to high chromophore density (3.09–4.02 × 10^20^ molecules cm^−3^). Long‐term alignment stability tests show that after heating at 85 °C for over 500 h, 99.73% of the initial *r*_33_ value is maintained for poled crosslinked electro‐optic films 1:1 FZL1/FZL2. The poled self‐assembled electro‐optic films 1:1 FZL1/FZL4 and 1:1 FZL3/FZL4 can still maintain more than 97.11% and 98.23%, respectively, of the original electro‐optic coefficient after being placed at room temperature for 500 h. The excellent electro‐optic coefficient and stability of the material indicate the practical application prospects of organic electro‐optic materials.

## Graphical abstract

Binary crosslink‐able/self‐assembled electro‐optical materials FZL1‐4 are successfully developed with high chromophore loading levels that possess ultrahigh electro‐optic coefficients and high long‐term alignment stability.

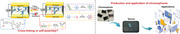

## 1 Introduction

Currently, with the rapid development of technologies such as cloud computing, 5G communication, high‐definition network video, terahertz field,^[^ ^1^ ^]^ artificial intelligence/machine learning (AI/ML), and the Internet of Things, the demand for information is growing rapidly without any slowdown.^[^ ^2^ ^]^With the rapid development of existing and the emergence of new types of services, and the demand for telework caused by the COVID‐19, the world has experienced an explosive growth in internet data traffic.^[^ ^3^ ^]^ There is a demand for ultra‐large capacity fiber optic communication in medium and short distance communication networks such as data center networks.^[^ ^4^ ^]^ For medium and short‐distance optical communication systems, how to achieve ultra‐high‐speed (single wavelength above 400Gb s^−1^) signal transmission in systems with limited bandwidth of optoelectronic devices has become a hot issue in the industry. To solve this problem, it is of great significance to study low‐cost single‐channel, high‐frequency spectral efficiency optical communication systems.

One of the key factors determining the application of optical communication technology is the preparation of high‐performance organic electro‐optical materials (second‐order nonlinear optical materials).^[^ ^5^ ^]^ The early studies on second‐order nonlinear optical materials were mostly inorganic crystal materials such as lithium niobate (LiNbO_3_).^[^ ^6^ ^]^ This type of material itself has a series of insurmountable shortcomings, such as low electro‐optical coefficient, difficulties in crystal growth and processing, high dielectric constant, and strong interference with the input light wave signal. After years of development, the advantages of organic electro‐optical materials have become increasingly apparent. Organic nonlinear optical materials have advantages such as high electro‐optic coefficient, fast response speed, and good processability and integration, making them widely used in fields such as electro‐optic modulators,^[^ ^7^ ^]^ optical communication, optical information storage, terahertz,^[^ ^8^ ^]^ etc. Moreover, it can be easily integrated with semiconductor microelectronic devices,^[^ ^9^ ^]^ thus having great application prospects.^[9a]^ The practical application of organic electro‐optic materials still faces many challenges. How to obtain organic electro‐optic chromophores with high electro‐optic coefficient (r_33_ value), photothermal stability, and poling orientation stability is still a research focus.^[^ ^10^ ^]^

So far, there are several common types of electro‐optic systems: guest/host material,^[^ ^11^ ^]^main chain polymer,^[^ ^12^ ^]^ side chain polymer,^[^ ^13^ ^]^ cross‐linked materials,^[^ ^14^ ^]^ hyperbranched dendritic molecules,^[^ ^15^ ^]^ self‐assembled molecular glass,^[^ ^16^ ^]^ etc. These systems each have their own unique characteristics, as well as their own advantages and disadvantages. The glass transition temperature is the temperature at which molecular order and electro‐optic activity are lost in organic nonlinear optical （NLO） materials.^[^ ^17^ ^]^ For integration with more complex devices, processing steps afterpoling may require high temperatures. So most host–guest doping systems, as well as systems with hyperbranched dendritic molecules were difficult to meet the practical needs due to the lower glass transition temperature （*T*_g_）^[^ ^18^ ^]^ Although the main chain and side chain polymers have high stability,^[^ ^19^ ^]^ they also have drawbacks: the low yield of chromophore attachment requires the synthesis of a large number of chromophores and the depoling temperature is significantly lower than *T*_g_ on a timescale of hours.^[^ ^20^ ^]^

One of the most influential and noteworthy organic electro‐optic material systems is cross‐linked organic electro‐optic materials, which can achieve high poling efficiency, large refractive index, good film forming ability, high chromophore density, and high film electro‐optic coefficient.^[^ ^21^ ^]^ The ordered arrangement of chromophores after poling can form an electro‐optic polymer network through cross‐linking reactions between molecules, immobilizing already oriented molecules and significantly improving the stability of the material.^[^ ^22^ ^]^ However, most cross‐linking systems occur between chromophores and polymers, and the content of chromophores is usually low, ≈25 wt.%, which limits the improvement of the electro‐optic coefficient.^[^ ^23^ ^]^ The electro‐optic coefficient of most cross‐linking systems is less than 150 pm V^−1^.^[^ ^24^ ^]^ The proposal of binary cross‐linked organic electro‐optical materials HLD1‐2^[^ ^25^ ^]^ and QLD1‐2^[^ ^26^ ^]^ changed this situation. The content of chromophores reaches 100 wt.%, and cross‐linking can occur between chromophores without the help of any polymers or small molecules. The electro‐optic coefficient is as high as 300 pm V^−1^, and the glass transition temperature is also greater than 180 °C. It effectively balances the electro‐optic coefficient and glass transition temperature, making it a highly promising organic electro‐optic material for practical use.

**Figure 1:**
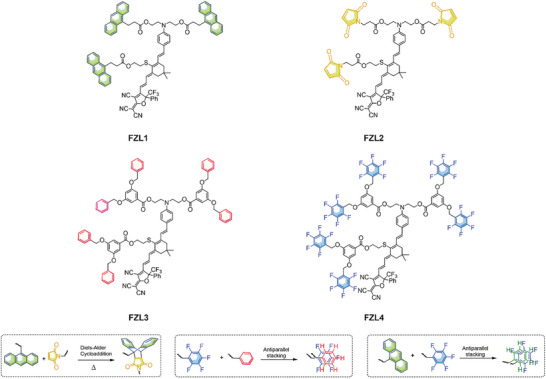
The Chemical Structure of Chromophores FZL1‐FZL4.

There is currently limited research on pure chromophore (without polymer or small molecule cross‐linking agent) cross‐linking systems, with most cross‐linked chromophores having only two cross‐linkable groups, usually on the donor and electron bridge or on the donor and acceptor.^[^ ^27^ ^]^ However, there was little research on chromophores with multiple cross‐linking groups.^[^ ^28^ ^]^ In addition, the cross‐linking method of binary cross‐linked organic electro‐optical materials (like HLD1‐2 and QLD1‐2) is limited to the DA reaction of anthracene and acrylate.^[^ ^29^ ^]^ This reaction requires 60 min of reaction at 160 °C to complete cross‐linking. Excessive temperature and longer reaction time may cause the decomposition of chromophores and the waste of energy. So it is very important to develop new cross‐linkable groups suitable for binary cross‐linked chromophores and study the effect of different quantities and positions of cross‐linkable groups on the performance of chromophores. For this reason, we designed chromophores FZL1‐FZL2 with three modifiable sites on the donor and electronic bridge, and three anthracene or maleimide groups were modified on the donor and electronic bridge of the chromophore to form a binary cross‐linked material, as shown in **Figure** **1**.

Moreover, the high cross‐linking temperature and long cross‐linking time of the current cross‐linking system are also limitations. The design and poling process of cross‐linking systems were very complex, while self‐assembled electro‐optical materials are relatively simple and also have strong poling orientation stability at room temperature although not at high temperature. So we designed chromophores FZL3‐FZL4 with three modifiable sites on the donor and electronic bridge, and three phenyl dendrons (HD) and pentafluorophenyl dendron (PFD) derivatives were modified on the donor and electronic bridge of the chromophore to form a binary self‐assembled material, which shown in Figure 1. Electro‐optic coefficient up to 272–308 pm V^−1^ was achieved in this electro‐optic film due to high chromophore density (3.09–4.02 × 10^20^ molecules cm^−3^) and large hyperpolarizability. Glass transition temperature as high as 178 °C was achieved by cross‐linked FZL1/FZL2. The poled and cross‐linked electro‐optic films 1:1 FZL1/FZL2 could still maintain more than 99.73% of the original electro‐optic coefficient being placed at 85 °C for 500 h. The poled electro‐optic films 1:1 FZL1/FZL4 and1:1 FZL3/FZL4 could still maintain more than 97.11% and 98.23%, respectively, of the original electro‐optic coefficient being placed at room temperature for 500 h. The series of FZL has both large electro‐optic coefficient and Long‐term alignment stability, which provides the possibility for the practical application of organic electro‐optic materials, and also provides a new idea for the design of cross‐linked/self‐assembly organic electro‐optic materials

## 2 Results and Discussion

### 2.1 Synthesis of Chromophores

**Figure 2:**
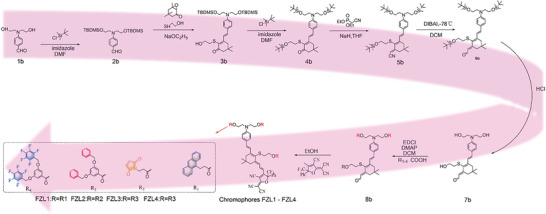
Synthetic Routes for Chromophores FZL1–4.

The chromophores FZL1‐4 have three functionalized groups, which require three OH groups on the donor and electron bridge of the chromophore. So the synthesis of chromophores involves the protection and deprotection of OH groups. The first step in the synthesis of chromophores was to protect the two hydroxyl groups on donor 1 with tert‐Butyldimethylsilyl chloride (TBDMS) groups to yield compound 2. The combination of the donor and bridge was achieved through the Knoevenagel condensation reaction to generate compound 3. Again, the dihydroxyl group on the bridge of the chromophores was protected by the TBDMS group to obtain compound 4. Trienenitrile 5 was obtained by Wittig–Horner reaction of compound 4 and diethyl(cyanomethyl)‐phosphonate under the catalysis of sodium hydride. The aldehyde 6 was achieved by the reduction with diisobutyl aluminium hydride (DIBAL‐H) of the nitrile group. Under acidic conditions, the three silanes on the donor and electron bridge were removed to form compound 7, facilitating subsequent modification of functionalized groups. Compound 8a–8d was obtained by Steglich esterification with 3‐(9‐anthracyl) propionic acid, 3‐Maleimidopropionic acid, 3,5‐bis(benzyloxy)benzoic acid and 3,5‐bis((perfluorophenyl)methoxy) benzoic acid. Finally, the aldehyde 8a–8d condensates with CF_3_‐TCF acceptor could obtain green solid chromophores FZL1‐4, as shown in **Figure** **2**.

### 2.2 Thermal Stability

**Figure 3:**
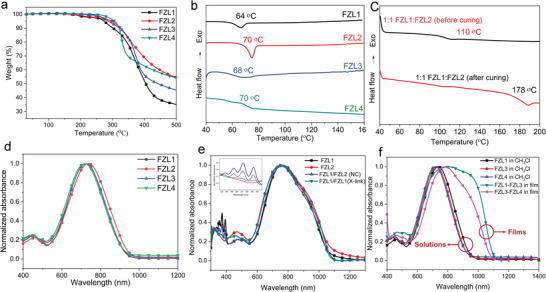
a) TGA curves of chromophores FZL1‐4. b) DSC curves of chromophores FZL1‐4. c) DSC curves of chromophores 1:1 FZL1:FZL2 before and after curing. d) The UV–vis absorption spectra of chromophores FZL1‐4 in the chloroform. e) The UV–vis absorption spectra of chromophores FZL1 and FZL2 in the thin films before and after cross‐linking f) The UV–vis absorption spectra of self‐assembled chromophores FZL1 and FZL3‐4 in the chloroform and films.

In order to measure the decomposition temperature (*T*_d_) and*T*_g_of the chromophores FZL1‐4, The thermal characteristics of the four chromophores were investigated using thermogravimetric analysis (TGA) and differential scanning calorimetry (DSC) under nitrogen as shown in **Table** **1** and **Figure** **3**a,c. All the chromophores exhibited good thermal stabilities with *T*_d_ higher than 270 °C. The *T*_g_’s of the chromophores FZL1‐4 was ranged from 64–70°C as shown in Figure 3b,c. These values were similar to similar chromophores with CF_3_‐TCF acceptor.^[^ ^30^ ^]^

**Table 1:** Thermal and optical properties data of the chromophores.

Cmpd	*T*_d_ [°C]	*T*_g_ [°C]	*λ*_max_^a)^	λ_max_^b)^	Δλ^c)^	λ_max_^d)^
FZL1	274	68	725	673	52	765
FZL2	298	70	738	669	69	751
FZL3	287	64	729	687	42	752
FZL4	307	70	718	680	38	724

^a,b,d)^(nm) was measured in chloroform, dioxane and in film, respectively;

^c)^ (nm) was the difference between ^a)^λ_max_ and ^b)^λ_max_.

Compared with the low *T*_g_ of the single chromophore, the blend of cross‐linkable chromophores provides a method for higher *T*_g_. The *T*_g_ of the 1:1FZ1/FZL2, rise to 110 °C, a low degree of cross‐linking can be achieved during the heating process in DSC measurement. But the heating time was limited, so, the degree of cross‐linking was not complete, leading to moderate *T*_g_. Then we tested the *T*_g_ of the chromophores 1:1FZ1/FZL2 after cross‐linking. During the heating process (135 °C for 30 min), the original small molecules were cross‐linked into polymers due to the Anthracene‐maleimideDA reaction, so after cross‐linking, The *T*_g_ of the 1:1FZ1/FZL2 mixture further rise to 178 °C. According to previous literature reports, for self‐assembled systems1:1FZ1/FZL4 and 1:1FZ3/FZL4, there will be no significant increase in the glass transition temperature after mixing.^[^ ^16b^ ^]^

### 2.3 Optical Properties

In order to compare the charge transfer ability and measuring the occurrence of cross‐linking and self‐assembly of chromophores FZL1‐4, we tested the UV–vis absorption spectra of chromophores FZL1‐4 and their blends in different solvents and thin films as shown in Table 1. The maximum absorption wavelengths of chromophores FZL1‐4 in chloroform ranges from 718 to 738 nm that indicates that the functionalized group hasn't changed the main conjugated structure of the molecule, which is shown in Figure 3d. Compared to chromophores FZL1‐3, using common aniline donor, the maximum absorption wavelength of the chromophore FZL4 was slightly blue‐shifted due to electron‐withdrawing effect of pentafluorobenzene. The solvatochromic behavior of the four chromophores in different solutions with different polarity and dielectric constant was studied as shown in Figure S9 (Supporting Information). The four chromophores FZL1‐4 exhibit similar π–π‐intramolecular charge transfer (ICT) absorption bands in non‐polar solvents such as 1,4‐dioxane. However, in more polar solvents including chloroform, dichloromethane, acetone, and acetonitrile a broad absorption band was also observed showing a continuous redshift red shift of *λ*_max_ along with a slight change of the spectral shape going from chloroform to acetonitrile. The four molecules showed broader absorption band in more polar solvents (such as chloroform and acetonitrile) compared to non‐polar solvents, and continuous redshift of *λ*_max_ was also observed. The positive solvatochromism and broad absorption band of chromophores FZL1‐4 in different solvents suggested that chromophores FZL1‐4 had neutral polyene‐like electronic structures in the ground state.^[^ ^31^ ^]^

The progression of cross‐linking state can be determined by ultraviolet absorption spectra before and after cross‐linking. The Individual chromophore FZL1 and FZL2 and their cross‐linked blend (1:1 FZL1/FZL2) were rotated into an electro‐optical film and tested for UV absorption as shown in Figure 3e. After curing (135 °C for 30 min), the strength of the anthracene absorption bands located near 350, 370, and 390 nm decreased significantly. It proves that a good efficiency of cross‐linking reaction between anthracene and maleimide of chromophores FZL1 and FZL2.^[^ ^32^ ^]^

The maximum absorption value of 1:1 FZL1/FZL4 mixture and 1:1 FZL3/FZL4 mixture was 805 and 778 nm in films, as shown in Figure 3f, respectively. Meanwhile, we can see that the mixed chromophore blends in the film were obviously red shift and have broader full width at half maxima (FWHM) compared to that in chloroform solution. Different absorption curves of chromophore molecules in thin films and solutions were mainly due to the inhomogeneous broadening and solvatochromism of the electronic absorption characteristics associated with chromophore–chromophore interactions.^[^ ^33^ ^]^ The intrinsic nature of the wilder peak appearing in the films of chromophores may due to the nanoscale acentric *J*‐aggregates between chromophores because of *π*–*π* stacking.^[^ ^31^ ^]^

### 2.4 Theoretical Calculations

**Figure 4:**
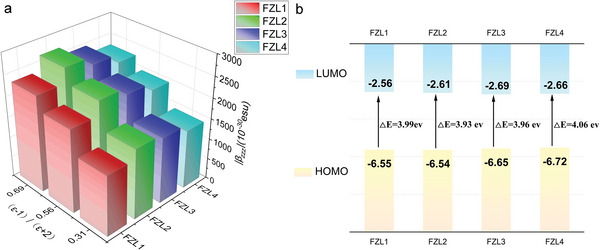
a) Hyperpolarizability in toluene, chloroform, and tetrahydrofuran solution for chromophores FZL1‐4 calculated by DFT (M062X/6‐31+G(d)). b) The energy level difference (ΔE) for the chromophores FZL1‐4.

By adopting Gaussian 09 software package, the Density functional method (DFT) theoretical calculations were carried out using the cam‐B3LYP method combined with the 6–31 g(d,p) basis group.^[^ ^34^ ^]^ Assumed that all chromophore molecules were trans‐structures, the optimized molecular structure, dipole moment, first‐order hyperpolarizability, and HOMO‐LUMO energy gap of chromophores FZL1‐4 were calculated.^[^ ^35^ ^]^ The electron cloud density and the ground and excited state energy levels of chromophores were shown in **Table** **2**. The HOMO‐LUMO energy gap (ΔE) of chromophores FZL1‐4 was ranged from 3.93 to 4.06 eV, as shown in **Figure** **4**b. The energy gap of these four chromophores was similar because their UV absorption was similar. Functionalized groups that can be cross‐linked/self‐assembled do not affect the conjugated structure of chromophores. The first‐order hyperpolarizability of the molecules in vacuum was calculated. The β value of FZL1‐4 range from 781.45 to 876.11 × 10^−30^esu, as shown in Table 2. The first‐order hyperpolarization of chromophore FZL4 was lower than that of FZL1‐3, which may be due to the electron absorption of pentafluorobenzene. The hyperpolarizability of chromophores FZL1‐3 was very close, because they have similar UV absorption wavelength and the same conjugate structure.^[^ ^36^ ^]^

**Table 2:** Summary of Optical Properties and DFT data.

Cmpd	ΔE[DFT]^a)^ [eV]	µ^b)^[D]	Cam ^c)^ [10^−30^esu]	Tetrahydrofuran [10^−30^esu]	Chloroform [10^−30^esu]	Toluene [10^−30^esu]
FZL1	3.99	22.30	866.88	2336	2011	1463
FZL2	3.93	23.54	876.11	2681	2338	1695
FZL3	3.96	22.53	860.19	2487	2178	1603
FZL4	4.06	23.00	781.45	2276	1983	1428

^a)^ was calculated from DFT calculations;

^b)^ was the total dipole moment;

^c)^ was the first‐order hyperpolarizability in vacuum calculated from DFT calculations.

In order to show solvent effects on hyperpolarizability and dipole moments of the chromophores FZL1‐4, we also calculated the first‐order hyperpolarizability of chromophores at the M062X/6‐31+G(d) level of theory in implicit solvent (PCM) environments in three different solvent: chloroform, tetrahydrofuran, and toluene, as shown in Figure 4a. Compared with the other two solvents, the first‐order polarizability of all chromophores in tetrahydrofuran solvent was the maximum, among which, the first‐order hyperpolarizability of FZL2 in tetrahydrofuran was the maximum, which was 2681 × 10^−30^ esu. In different solvents, FZL1‐4 also achieved similar β value, of which FZL4 was the smallest, which was consistent with the calculation results in vacuum.

### 2.5 Testing of Electro‐Optical Coefficients

In order to test the electro‐optic coefficient and poling efficiency of chromophores, we prepared an electro‐optic thin film using chromophores FZL1‐FZL4 and their blends. Chromophores FZL1‐FZL4, 1:1 FZL1/FZL2, 1:1 FZL1/FZL4 and 1:1 FZL3/FZL4 were dissolved in newly distilled dibromomethane (ultrasound for half an hour), filtered by 0.2 mm Teflon needle filter and then coated on an indium oxide (ITO) glass at optimal speed to form an electro‐optic thin film with a thickness of approximately 1000 nm. The individual FZL1‐FZL4 and 1:1 FZL3/FZL4 film was dried overnight at 50 °C in the vacuum overnight to remove the residual solvents. The 1:1 FZL1/FZL2 film was dried at 65 °C for 12 h for the pre‐cross‐linking process. The benzocyclobutene (BCB) charge injection barrier layer was for some device to avoid current leakage.^[^ ^37^ ^]^ The poling process is carried out under the optimal temperature and electric field. After polarization is completed, the electro‐optic film gradually cools to room temperature while maintaining the electric field. The electro‐optic coefficient (*r*_33_) of the poled film was measured at 1310 nm using Teng‐man simple reflection technique, as shown in Figure 6a.^[^ ^38^ ^]^

If the electrostatic interactions between molecules can be ignored, the electro‐optic coefficient of the chromophore is proportional to the number density of the chromophore, the first‐order hyperpolarization of the molecule, and the poling electric field.^[^ ^39^ ^]^ However, chromophore molecules have significant dipole–dipole interactions, which can hinder the molecular rotation under the action of an electric field. So introducing steric hindrance groups into chromophores can effectively improve the poling efficiency of chromophores.^[^ ^40^ ^]^

**Figure 5:**
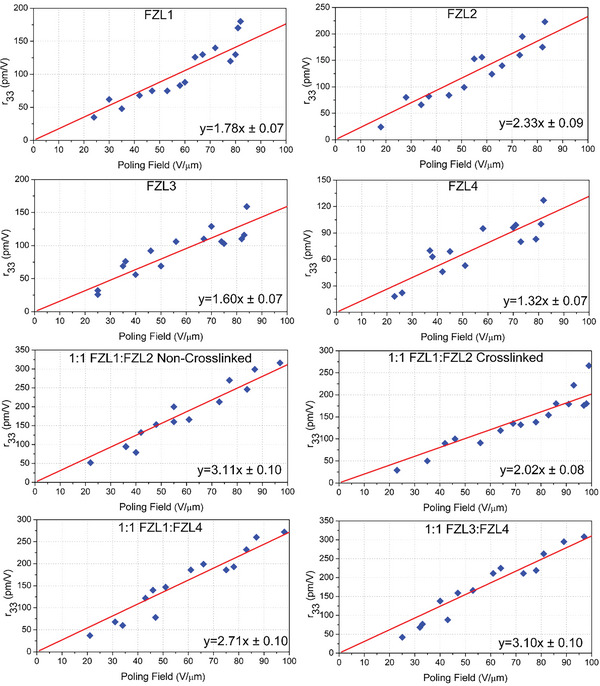
Poling curve plots of *r*_33_ versus poling field.

**Figure 6:**
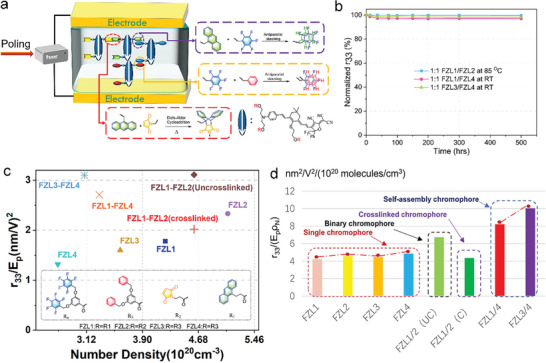
a) The cross‐linking process of chromophore molecules FZL1‐4. b) Temporal stability of the poled films FZL1/FZL2, FZL1/FZL4, and FZL3/ FZL4 at the temperature of 85 °C or room temperature in vacuum. c) The *r*_33_/*E*_p_ value of chromophores FZL1–4 and their mixtures as a function of N. d) The *r*_33_/(*E*_p_ρ_N_) value of individual chromophores FZL1‐FZL4 and their blends.

The electro‐optical performance of the individual chromophores was tested. The poling process was a conventional method with temperature 5–10 °C higher than the *T*_g_ of the chromophore. The maximum *r*_33_ value of chromophores FZL1‐FZL4 ranges from 127 to 223 pm V^−1^. The average poling efficiencies (*r*_33_/*E*_p_) of FZL1‐FZL4 range from 1.32 ± 0.07 to 2.33± 0.09 nm^2^ V^−2^ as shown in **Figure** **5** and **Table** **3**. According to the calculation results of UV and DFT theory, the four chromophores FZL1‐FZL4 have similar first‐order hyperpolarization due to the same conjugate structure. The difference in *r*_33_ value and average poling efficiencies was mainly due to the different number density of chromophores as shown in **Figure** **6**c. The chromophore FZL2 achieves the maximum electro‐optical coefficient and polarization efficiency because it has the highest density of chromophores (5.09 × 10^20^ molecules cm^−3^). The smaller electro‐optical coefficients of chromophores FZL3 and FZL4 were also due to the decrease in the number density (2.72–3.59 × 10^20^ molecules cm^−3^) of chromophores due to their high molecular weight.

**Table 3:** Electric Field Poling Data for EO Chromophores in Bulk Devices.

Cmpd	ρ_N_ ^a)^	*r*_33_/*E*_p_ [nm^2^/V^2^]^b)^	*r*_33_/[*E*_p_ *ρ*_N_]^c)^	max.*r*_33_ [pm/V]
FZL1	4.22	1.78 ± 0.07	4.22 ± 0.17	180
FZL2	5.09	2.33 ± 0.09	4.57 ± 0.18	223
FZL3	3.59	1.60 ± 0.07	4.46 ± 0.19	159
FZL4	2.72	1.32 ± 0.07	4.85 ± 0.26	127
1:1 FZL1‐FZL2(Uncross‐linked)	4.62	3.11 ± 0.10	6.73 ± 0.22	316
1:1FZL1‐FZL2(Crosslinked)	4.62	2.02 ± 0.08	4.37 ± 0.17	266
1:1FZL1‐FZL4	3.30	2.71 ± 0.10	8.21 ± 0.30	272
1:1FZL3‐FZL4	3.09	3.10 ± 0.10	10.03 ± 0.32	308

^a)^ Number density (assumes mass density of 1 g cm^−3^, in unit of × 10^20^ molecules cm^−3^);

^b)^ Average from multiple poling experiments;

^c)^ Poling efficiency per number density (nm^2^ V^−2^(10^20^ molecules cm^−3^)^−1^).

We then mix the chromophore FZL1‐FZL4 in pairs to form a binary chromophore system. When the poling temperature does not reach the cross‐linking reaction temperature, the binary system can be regarded as an ordinary binary chromophore. The average *r*_33_/*E*_p_ values of 1:1 FZL1‐FZL2, 1:1 FZL1‐FZL4 and 1:1 FZL3‐FZL4 were 3.11± 0.10, 2.71 ± 0.10, and 3.10 ± 0.10 nm^2^ V^−2^, as shown in Figure 5, respectively. The poling efficiency of binary systems far exceeds that of single component chromophores, which also indicates that binary mixing helps to improve the electro‐optical coefficient of chromophores. It is worth pointing out that the *r*_33_/(*E*_p_ρ_N_) value of self‐assembled binary material 1:1 FZL1‐FZL4 and 1:1 FZL3‐FZL4 was larger than that of uncross‐linked 1:1 FZL1‐FZL2, as shown in Figure 6d. The π–π stacking of pentafluorobenzene‐anthracene and pentafluorobenzene ‐benzene could help to obtain highly poling‐induced order. In addition to the binary chromophore effect, the characteristics of self‐assembled materials together create large *r*_33_/(*E*_p_ρ_N_) value. The maximum *r*_33_ value of chromophores 1:1 FZL1‐FZL2, 1:1 FZL1‐FZL4, and 1:1 FZL3‐FZL4 ranges from 272 to 316 pm V^−1^, which was one of the highest values reported, as shown in Table 3.

The poling process of cross‐linked 1:1 FZL1‐FZL2 requires higher temperatures (135 °C). Before cross‐linking, the chromophore is placed in a vacuum drying oven at 65 °C for 12 h for pre‐cross‐linking to allow higher voltage loading. In order to prevent the film from cracking, a step poling method was used: under desired poling field loading, the film was heated to 110 °C at 10 °C min^−1^ and held for 10 min, heated to 120 °C and held for 10 min, then heated to 135 °C and held for 10 min, and then cooled to room temperature. With the help of the cross‐linking reaction, the allowable loading poling field increased to ≈100 V m^−1^. After cross‐linking, the electro‐optical coefficients of cross‐linked 1:1 FZL1‐FZL2 were 266 pm V^−1^. This value was smaller than uncross‐linked 1:1 FZL1‐FLZ2 that is also easy to understand because cross‐linking process can hinder poling to some extent. However, the electro‐optic coefficient of close to 300 pm V^−1^ was still one of the highest reported values.

In order to achieve practical and commercial applications of materials, it is necessary to maintain long‐term stability in their performance. Long‐term and/or high‐temperature alignment stability test was conducted as shown in Figure 6b. The poled and cross‐linked electro‐optic films 1:1 FZL1/FZL2 could still maintain more than 99.73% of the original electro‐optic coefficient being placed at 85 °C for 500 h, as shown in Figure 6b. The poled electro‐optic films 1:1 FZL1/FZL4 and1:1 FZL3/FZL4 could still maintain more than 97.11% and 98.23%, respectively, of the original electro‐optic coefficient being placed at room temperature for 500 h. The above results indicate that the high glass transition temperature of the binary cross‐linking system after cross‐linking and the self‐assembled chromophore is beneficial for the long‐term alignment stability of the chromophore.

### 2.6 Comparison and Future Prospects of Different Cross‐linking Systems

Currently, there were very few reports on binary neat (100 wt.% chromophore) cross‐linked chromophores, and the types of cross‐linking reactions were limited to the DA reaction between anthracene and acrylate. The temperature of traditional anthracene‐acrylate cross‐linking can reach up to 160 °C, and the cross‐linking time also exceeds an hour. In order to shorten the temperature and time of cross‐linking reaction and improve the poling efficiency, we designed chromophores FZL1‐2 containing three anthracene and maleimide groups on the donor and electronic bridge. The results show that the glass transition temperature and electro‐optic coefficient of Chromophores FZL1‐2 (266 pm V^−1^ and 178 °C) were equivalent to those of chromophores HLD1‐2 (290 pm V‐^1^ and 174 °C) and FLD1‐2 (327 pm V^−1^ and 185 °C) when the poling and cross‐linking temperature (135 °C) was lower and the cross‐linking time was shorter (30 min), as shown in Table S2 (Supporting Information). The difference in glass transition temperature of FZL1‐2 may be due to the varying number of cross‐linking groups, resulting in varying degrees of cross‐linking. The electro‐optic coefficient of chromophores FZL1‐2 is slightly smaller than that of HLD1‐2 and FLD1‐2 because of the decrease of Number density of chromophore (4.62 vs 5.14 and 4.81 × 10^20^ molecules cm^−3^) caused by more branched groups. However, from this perspective, the AM‐DA reaction is also a very effective reaction for improving the *T*_g_ and of binary neat cross‐linked chromophores system. And the lower cross‐linking temperature of Anthracene‐maleimide‐based Diels–Alder (DA) reaction and shorter cross‐linking time are beneficial for accelerating the poling process and improving the poling efficiency.

Except for Anthracene‐acrylate‐based and Anthracene‐maleimide‐based Diels–Alder (DA) reaction, Maleimide‐furan‐based Diels–Alder reaction and Azide‐alkyne‐based Huisgen cycloaddition reaction, Anthracene‐anthracene photocross‐linking and thiol‐Michael reactions may also be used to construct binary cross‐linking systems in the future. The biggest obstacle to the commercialization of organic electro‐optical materials is their weak stability. The *T*_g_ is the temperature at which molecular order and electro‐optic activity are lost in organic NLO materials. Telcordia standards require operation at 85 °C for 2000 h. So, *T*_g_ must be well above this threshold to meet this goal. For integration with more complex devices, processing steps after EO poling may require high temperatures. So, thermally stable EO activity at 200 °C would be hugely important. At present, the maximum *T*_g_ of binary cross‐linked electro‐optic materials is only 185 °C, which has not yet reached the ideal standard of over 200 °C. Perhaps the *T*_g_ of the chromophore can be improved by introducing rigid cross‐linking groups, such as the bis‐anthracene structure. In addition to stability, a simpler cross‐linking formula is also very important, as the design of binary cross‐linking chromophores involves issues of ratio and chromophore matching. Perhaps integrating crosslink‐able groups into a single chromophore to form a single component crosslink‐able electro‐optic material system is more conducive to industrial production.

## 3 Conclusion

In order to design and synthesize organic electro‐optic materials with high electro‐optic coefficient and long‐term stability, we developed a series of isophorone‐based highly efficient chromophores FZL1‐4 with three different cross‐linkable/ self‐assembled groups modified on the donor and bridge part of the molecules. The oriented molecules were fixed by covalently or non‐covalently cross‐linked network that formed by Anthracene‐maleimideDAreaction or Anthracene‐pentafluorobenzene and benzene‐pentafluorobenzene π–π interaction after electric field poling orientation. It could significantly improve the long‐term alignment stability of the materials. The 100 wt.% chromophore content creates a large electro‐optic coefficient of the chromophore FZL1‐4. After poling and cross‐linking or self‐assembled process, the cross‐linked film FZL1/FZL2 and self‐assembled films FZL1/FZL4 and FZL3/FZL4 films achieved very high maximum *r*_33_ values of 266, 272, and 308 pm V^−1^, respectively, due to high chromophore density (3.09–4.02 × 10^20^ molecules cm^−3^). Long‐term alignment stability tests showed that after heating at 85 °C for over 500 h, 99.73% of the initial *r*_33_ value was maintained for poled cross‐linked electro‐optic films 1:1 FZL1/FZL2. After being annealed at room temperature, 97.11% and 98.23% of the initial *r*_33_ value of self‐assembled electro‐optic films 1:1 FZL1/FZL4 and1:1 FZL3/FZL4 could be maintained for over 500 h, respectively. The excellent electro‐optic coefficient and stability of the material indicate the practical application prospects of organic electro‐optic materials. These results provide valuable references for the design of cross‐linked materials with both large electro‐optic coefficient and high glass transition temperature, and provide possibilities for the commercialization and device application of organic electro‐optic materials.

## Conflict of Interest

The authors declare no conflict of interest.

## Supporting information

Supporting Information

## Data Availability

The data that support the findings of this study are available in the supplementary material of this article.
