# Supplementary material for: Highly Efficient and Stable Binary Cross‐Linkable/ Self‐Assembled Organic Nonlinear Optical Molecular Glasses
Source: Adv Sci (Weinh). 2023 Sep 10;10(31):2304229. doi: 10.1002/advs.202304229 (PMC10625134; doi:10.1002/advs.202304229)
Supplement: Supplementary file 1 — Supporting Information [file ADVS-10-2304229-s001.pdf]

ADVANCED SCIENCE
Open Access

Supporting Information

for *Adv. Sci.*, DOI 10.1002/advs.202304229

Highly Efficient and Stable Binary Cross-Linkable/ Self-Assembled Organic Nonlinear Optical Molecular Glasses

Lian Zhang, Fenggang Liu, Ruoxi Yang, Fuyang Huo, Weijun Zhang, Yu Zhang, Chuying Liu, Chunngai Hui and Jiahai Wang**

Supporting Information

Highly Efficient and Stable binary cross-linkable/ self-assembled organic nonlinear optical molecular glasses

Lian Zhang [a], Fenggang Liu [a]*,Ruoxi Yang [a] , Fuyang Huo [a], Weijun Zhang [a], Yu Zhang [a], Chuying Liu [a], Chunngai Hui b and Jiahai Wang [a] *

a School of Chemistry and Chemical Engineering, Guangzhou University, Guangzhou 510006, P. R. China. E-mail: liufg6@gzhu.edu.cn; jiahaiwang@gzhu.edu.cn

b Huawei Technoligies, Bantian Industrial Base, Shenzhen 518129, P. R. China.

Table of Contents

1.Materials and instruments.. 2

2. Experimental 2

3. NMR pictures.. 7

4. UV-Vis Absorption Spectroscopy.. 12

5. Differential Scanning Calorimetry testing.. 12

6. DFT Calculations.. 15

7. Properties of the state-of-the-art organic EO materials. 16

8. Comparison of different crosslinking systems. 17

9. Electric field induced polarization 18

10. Long-term alignment stability test.... 18

11. Reference.. 18

1. Materials and instruments

The chemicals used in this paper were commercially available and do not require further purification unless otherwise stated. The solvents used in the experiment like tetrahydrofuran (THF), N,N-dimethylformamide (DMF) and dichloromethane(DCM) were commercial ultra-dry reagents. Thin-layer chromatography on 0.25 mm-thick pre-coated silica gel plates and showed spots under UV light. Kieselgel (60-100 mesh and 200-300 mesh) silica gel chromatography was used.

The specific synthesis steps of chromophores FZL1-4 and its intermediates and the characterization data of mass spectrum, hydrogen spectrum and carbon spectrum are shown in the supporting information.

^{1}H-NMR and ^{13}C-NMR spectra were obtained by an Advance Bruker 400M (400 MHz) NMR spectrometer (tetramethyl silane was used as an internal reference). Mass spectra were obtained on a MALDITOF (matrix-assisted laser desorption/flight ionization). BIFLEX III (Broker Inc.) spectrometer. UV-Vis spectra were performed on a Cary 5000 spectrometer. TGA was determined by TA5000-2950TGA (TA co) with a heating rate of 10 °C min^{-1}, under nitrogen protection. Glass-transition temperature (T_g) was measured by DSC8000 (Perkin Elmer) with a heating rate of 10 °C min^{-1} under the protection of nitrogen.

2 Experimental

2.1 Synthesis of compound 2b

In a two-neck round bottom flask, imidazole (4.09g, 60mmol) and t-Butyldimethylchlorosilane (9.04 g, 60 mmol) were placed, then vacuumed and backfilled with argon gas, which was repeated three times. After that, N, N-dimethylformamide was slowly injected into the flask to dissolve 4-[N, n-bis (2-hydroxyethyl) amino] benzaldehyde (5 g, 24 mmol). After 3 h of reaction at room temperature, 100 ml water was poured into the reaction mixture, the product was then extracted by ethyl acetate for three times, finally $MgSO_4$ was added to remove excess water. the solvent was removed in a vacuum, Flash chromatography of the crude (acetone: hexane = 1:15 to 1:10) over SiO_2 to obtained oily compound 2b (10.1 g, 23.1mmol) with a yield of 96%. MS (MALDI) (M+, C23H43NO3Si2): calcd: 437.27; found: 437.30.^{1}H NMR (600 MHz, CDCl3) δ 9.70 (s, 1H), 7.69 (d, J = 8.8 Hz, 2H), 6.74 (d, J = 8.9 Hz, 2H), 3.80 (t, J = 6.1 Hz, 4H), 3.62 (t, J = 6.1 Hz, 4H), 0.87 (s, 18H), 0.02 (s, 12H).

2.2 Synthesis of compound 3b

Under ice bath condition, Slowly dissolve sodium metal (0.64 g, 27.72 mmol) in 30 mL of ethanol, The above reaction occured in a 500 mL two-mouth flask filled with argon gas. Then add 2-mercaptoethanol (2.17 g, 27.72 mmol) to the above solution. After 20 min of reaction at room temperature, compound 2b (10.1 g, 23.1mmol) was added. Then, Stired with a magnetic mixer for 1 h, the reaction heated at 65 ℃ to reflux for overnight. Added MgSO4 to mixture to remove water, and stripped of solvent by rotary evaporator. The crude product was ethyl acetate and n-hexane (1:10~1:4) as eluent, and compound 3b (11.8 g, 18.6mmol) was obtained by column chromatography with a yield of 81%. MS (MALDI) (M+, $C_{34}H_{59}NO_4SSi_2$):calcd: 633.37; found: 633.40.^{1}H NMR (600 MHz, $CDCl_3$) δ 7.89 (d, *J* = 16.0 Hz, 1H), 7.44 (d, *J* = 7.6 Hz, 2H), 7.02 (d, *J* = 16.0 Hz, 1H), 6.69 (d, *J* = 7.6 Hz, 2H), 4.15 – 4.11 (m, 2H), 4.09 (td, *J* = 6.8, 1.5 Hz, 2H), 3.79 (t, *J* =

5.8 Hz, 4H), 3.57 (t, *J* = 5.8 Hz, 4H), 3.47 (s, 1H), 3.00 (td, *J* = 6.5, 1.6 Hz, 2H), 2.95 (ddd, *J* = 15.0, 6.9, 1.8 Hz, 2H), 1.10 (d, *J* = 1.2 Hz, 6H), 0.93 – 0.92 (m, 18H), 0.14 – 0.07 (m, 12H).

2.3 Synthesis of compound 4b

In a 500 mL round bottom flask were combined imidazole (1.52 g, 22.3 mmol) and t-Butyldimethylchlorosilane (3.36 g, 22.3 mmol), then, added to compound 3b (11.8 g, 18.6mmol) dissolved in 30ML of N, N-dimethylformamide. The solution was stirred at room temperature under argon atmosphere for 3 hours, and then pour 100 mL of water, The solution was filtered to remove the water and MgSO4, and stripped of solvent by rotary evaporator. After removing the solvent in vacuum, the crude product was purified by silica gel chromatography and eluted with ethyl acetate /n-hexane (1:15~1:10) to obtain compound 4b (12.36 g, 16.5 mmol) with a yield of 89%. MS (MALDI) (M+, $C_{40}H_{73}NO_4Si_3$):calcd: 747.45; found:748.30. ^{1}H NMR (600 MHz, $CDCl_3$) δ 7.91 (d, *J* = 16.2 Hz, 1H), 7.45 (d, *J* = 8.9 Hz, 2H), 7.00 (d, *J* = 16.2 Hz, 1H), 6.69 (d, *J* = 8.9 Hz, 2H), 4.13 (q, *J* = 7.1 Hz, 2H), 3.83 – 3.76 (m, 4H), 3.71 (t, *J* = 7.1 Hz, 2H), 3.58 (t, *J* = 6.3 Hz, 4H), 2.89 (t, *J* = 7.1 Hz, 2H), 2.62 (s, 2H), 1.09 (s, 6H), 0.95 – 0.90 (m, 27H), 0.12 – 0.10 (m, 18H).^{13}C NMR (151 MHz, $CDCl_3$) δ 195.89, 157.88, 149.07, 137.21, 129.43, 128.08, 124.10, 123.48, 111.62, 62.90, 60.26, 53.49, 51.97, 51.83, 41.30, 36.47, 32.29, 28.39, 28.11, 25.92, 25.90, 25.66, 18.27, 17.99, -3.58, -5.23, -5.35.

2.4 Synthesis of compound 5b

Compound 4b (12.36 g, 16.5 mmol), diethyl cyanomethyl phosphate (11.69 g, 66mmol) , sodium hydride (1.58 g, 66mmol) were added to 40 mL tetrahydrofuran in the flask, The above reaction under the protection of argon and ice bath condition. After the solution is clarified , the reaction was heated at 65 ℃ to reflux for overnight. After tetrahydrofuran was removed in vacuum, it was directly purified with ethyl acetate /n-hexane (1:15~1:10) silica gel elution column chromatography to obtain red compound 5b (8.91 g, 11.55 mmol) with 70% yield. MS(MALDI) (M+,$C_{42}H_{75}NO_4SSi_3$): calcd: 773.47; found: 773.50. ^{1}H NMR (600 MHz, $CDCl_3$) δ 10.16 (d, *J* = 8.1 Hz, 1H), 7.97 (d, *J* = 16.2 Hz, 1H), 7.42 (d, *J* = 8.8 Hz, 2H), 7.02 (d, *J* = 8.1 Hz, 1H), 6.87 (d, *J* = 16.2 Hz, 1H), 6.69 (d, *J* = 8.9 Hz, 2H), 3.79 (t, *J* = 6.3 Hz, 4H), 3.71 (t, *J* = 7.2 Hz, 2H), 3.57 (t, *J* = 6.3 Hz, 4H), 2.76 (s, 2H), 2.72 (t, *J* = 7.2 Hz, 2H), 2.52 (s, 2H), 1.06 (s, 6H), 0.91 (d, *J* = 4.6 Hz, 18H), 0.88 (s, 9H), 0.05 (s, 12H), 0.04 – 0.02 (m, 6H). ^{13}C NMR (151 MHz, $CDCl_3$) δ 191.60, 156.66, 150.11, 148.64, 134.74, 128.96, 128.29, 126.92, 124.74, 111.65, 62.61, 60.28, 53.52, 41.68, 39.93, 37.52, 30.07, 28.34, 25.91, 18.33, 18.28, 14.21, -5.25, -5.34.

2.5 Synthesis of compound 6b

Under the protection of argon gas, slowly add diisobutyl aluminium hydride solution (2.74 mL, 23.1 mmol) to 80.0 mL fresh dry toluene solution of compound 5b (8.91 g, 11.55 mmol) that had been precooled in a low-temperature reactor at - 78 ℃ for 2h, then, add 10 g of wet silica gel and 15.0 mL of water, and continued the reaction at 0 ℃ for 2h. Water was poured into the reaction product and extract the crude product with ethyl acetate. The crude product was purified by vacuum concentration and silica gel chromatography column, and eluted with ethyl acetate /n-hexane (1:15~1:10) solution. Finally, red solid compound 6b (6.27 g, 8.1 mmol) with a yield of 70.1%. MS

(MALDI)(M+, $C_{24}H_{33}NO_4S$): calcd: 431.21; found: 431.40.^{1}H NMR (600 MHz, $CDCl_3$) δ 10.15 (d, *J* = 8.0 Hz, 1H), 7.46 (dd, *J* = 8.9, 2.2 Hz, 2H), 7.01 (d, *J* = 8.0 Hz, 1H), 6.91 (d, *J* = 16.1 Hz, 1H), 6.72 (t, *J* = 8.9 Hz, 2H), 6.55 (dd, *J* = 15.7, 7.9 Hz, 1H), 3.92 (dt, *J* = 9.6, 4.9 Hz, 4H), 3.68 (dt, *J* = 17.9, 4.7 Hz, 6H), 3.55 (s, 1H), 3.37 (s, 2H), 2.78 (dd, *J* = 10.5, 4.4 Hz, 2H), 2.54 (s, 4H), 1.06 (s, 6H).^{13}C NMR (151 MHz, $CDCl_3$) δ 193.88, 153.61, 135.04, 128.92, 127.83, 126.86, 124.22, 122.49, 112.36, 61.18, 60.72, 60.56, 41.74, 39.91, 38.18, 30.12, 28.31.

2.6 Synthesis of compound 7b

Added 30 mL HCl with concentration of 1M to a flask contained compound 6b (6.27 g, 8.1 mmol) and 50 mL acetone. After stirred at room temperature for 3 hours, neutralized with sodium bicarbonate, and used vacuum drying oven for vacuum concentration. 50 mL water was poured into the concentrated solution, extracted it with 100 mL dichloromethane for three times. The obtained product was concentrated in vacuum, and the water was removed with MgSO4. The crude product was purified by silica gel chromatography column and eluted with dichloromethane /ethyl acetate (10:1~1:1) gradient to obtain red solid compound 7. MS(MALDI) (M+ $C_{75}H_{69}NO_7S$): calcd: 1127.47; found: 1127.50. ^{1}H NMR (600 MHz, $CDCl_3$) δ 10.20 (dd, *J* = 19.0, 8.0 Hz, 1H), 8.36 (dt, *J* = 25.2, 8.8 Hz, 2H), 8.22 (dd, *J* = 16.2, 8.6 Hz, 4H), 7.97 (ddt, *J* = 20.4, 13.6, 11.6 Hz, 5H), 7.73 (d, *J* = 8.2 Hz, 1H), 7.64 – 7.50 (m, 5H), 7.50 – 7.35 (m, 7H), 7.30 – 7.18 (m, 6H), 7.03 (dd, *J* = 22.0, 8.0 Hz, 1H), 6.87 (ddd, *J* = 16.8, 12.5, 7.6 Hz, 1H), 6.73 (ddd, *J* = 19.8, 8.8, 4.9 Hz, 1H), 6.63 (ddd, *J* = 16.8, 14.4, 7.7 Hz, 1H), 6.59 – 6.53 (m, 1H), 4.26 – 4.06 (m, 4H), 3.98 – 3.86 (m, 4H), 3.41 (dddd, *J* = 22.4, 17.6, 13.8, 7.5 Hz,4H), 2.88 – 2.71 (m, 7H), 2.64 – 2.46 (m, 2H), 2.33 – 2.22 (m, 2H), 2.08 (s, 4H), 1.08 (d, *J* = 7.2 Hz, 6H).^{13}C NMR (151 MHz, $CDCl_3$) δ 191.58, 172.90, 171.18, 156.19, 139.51, 139.39, 136.14, 134.86, 133.76, 133.04, 132.35, 132.14, 131.58, 129.49, 129.38, 129.28, 129.03, 127.98, 127.12, 126.51, 126.31, 126.03, 125.37, 125.20, 124.98, 123.85, 111.97, 63.02, 61.38, 60.44, 49.08, 46.28, 41.73, 39.90, 35.17, 34.06, 33.33, 32.25, 31.99, 30.82, 30.13, 29.75, 28.37, 26.96, 23.69, 23.31, 23.18, 22.79, 21.10, 14.26.

2.7 Synthesis of compound 8a

3 - (anthracene-9-yl) propionic acid (1.30 g, 5.22 mmol), 4-dimethylaminopyridine (0.063 g, 0.52 mmol), 1-Ethyl-3-(3-dimethylaminopropyl) carbodiimide hydrochloride (1.00 g, 5.22 mmol) were added to 20 mL dichloromethane in a 100 ml double neck flask filled with argon gas. After reacted at 0 ℃ for 1 hour, the solution gradually became clear, then compound 7b (0.50 g, 1.16 mmol) dissolved in 10 mL dichloromethane were added, after that, the reaction above continued at 0 ℃ for 2 hours. The flask was then removed from the ice bath, the mixture was refluxed overnight at 65℃. 100 ml water was poured into the reaction mixture, the product is then extracted by ethyl acetate for three times, finally MgSO4 is added to remove excess water. the solvent was removed in a vacuum, Flash chromatography of the crude (acetone: hexane = 1:8 to 1:1) over SiO2 to obtain red oily compound 8a (0.79 g, 0.70 mmol) was 60%, MS(MALDI) (M+, $C_{87}H_{81}NO_{13}S$): calcd: 1380.65; found: 1380.55. ^{1}H NMR (600 MHz, $CDCl_3$) δ 10.02 (d, *J* = 8.0 Hz, 1H), 7.79 (d, *J* = 16.1 Hz, 1H), 7.27 – 7.18 (m, 21H), 7.18 – 7.15 (m, 6H), 7.15 – 7.11 (m, 5H), 7.09 (d, *J* = 2.1 Hz, 5H), 6.89 (d, *J* = 7.9 Hz, 1H), 6.66 (d, *J* = 2.0 Hz, 1H), 6.62 (d, *J* = 6.1 Hz, 3H), 6.58 (d, *J* = 8.0 Hz, 2H), 4.82 (d, *J* = 21.8 Hz,

12H), 4.29 (dd, *J* = 36.3, 30.7 Hz, 5H), 4.20 (t, *J* = 6.2 Hz, 2H), 3.59 (d, *J* = 5.3 Hz, 4H), 2.77 (t, *J* = 6.1 Hz, 2H), 2.57 (s, 2H), 2.23 (s, 2H), 0.88 (s, 6H). ^{13}C NMR (151 MHz, $CDCl_3$) δ 191.59, 171.18, 166.19, 165.92, 159.88, 159.83, 156.14, 150.97, 148.18, 136.54, 136.52, 134.83, 131.97, 131.68, 129.16, 128.70, 128.68, 128.18, 128.16, 127.71, 127.64, 127.62, 127.60, 127.11, 125.83, 125.20, 112.26, 108.58, 108.43, 107.62, 107.37, 70.26, 63.63, 62.19, 60.46, 49.49, 30.09, 28.42, 21.13, 14.32.

2.8 Synthesis of compound 8b

Using compound 7a and 3-Pyrroline-1-propionicacid as reactants, according to the reaction procedure of compound 8a, to obtain red oily compound 8b (0.72 g, 0.81 mmol) was 70%. MS(MALDI) (M+, $C_{45}H_{48}N_4O_{13}S$): calcd: 884.95; found: 884.86. ^{1}H NMR (600 MHz, $CDCl_3$) δ 10.13 (d, *J* = 8.0 Hz, 1H, CHO), 7.92 (d, *J* = 16.2 Hz, 1H, CH), 7.41 (d, *J* = 8.7 Hz, 2H, ArH), 6.94 (d, *J* = 8.0 Hz, 1H), 6.86 (d, *J* = 16.2 Hz, 1H, CH), 6.74 (d, *J* = 8.8 Hz, 2H, ArH), 6.68 (d, *J* = 11.8 Hz, 6H, CH), 4.25 (t, *J* = 6.2 Hz, 4H, NCH2), 4.10 (t, *J* = 7.1 Hz, 2H, NCH2), 3.80 (t, *J* = 6.7 Hz, 4H, NCH2), 3.75 (t, *J* = 7.0 Hz, 2H, OCH2), 3.65 (t, *J* = 6.1 Hz, 4H, OCH2), 2.78 (t, *J* = 6.5 Hz, 2H, SCH2), 2.63 (t, *J* = 7.0 Hz, 4H, CH2), 2.59 (t, *J* = 7.0 Hz, 2H, CH2), 2.51 (s, 2H, CH2), 2.03 (s, 2H, CH2), 1.04 (s, 6H, CH3). ^{13}C NMR (151 MHz, $CDCl_3$) δ 191.57, 170.65, 170.44, 170.38, 156.20, 150.79, 147.90, 134.78, 134.22, 129.05, 127.60, 127.02, 125.77, 125.21, 112.11, 63.09, 61.63, 60.38, 49.41, 41.65, 39.82, 33.55, 33.20, 32.90, 30.08, 28.30, 21.04, 14.19.

2.9 Synthesis of compound 8c

Using compound 7b and 3,5-bis (benzyloxy)benzoic acid as reactants, according to the reaction procedure of compound 8a, to obtain red oil like compound 8c (1.19 g, 0.86 mmol) was obtained in 74% yield. MS（MALDI）(M+,$C_{87}H_{81}NO_{12}S$)：calcd: 1363.55; found: 1363.45. ^{1}H NMR (600 MHz, $CDCl_3$) δ 10.02 (d, *J* = 8.0 Hz, 1H), 7.79 (d, *J* = 16.1 Hz, 1H), 7.27 – 7.18 (m, 5H), 7.18 – 7.15 (m, 4H), 7.09 (d, *J* = 2.1 Hz, 1H), 6.89 (d, *J* = 7.9 Hz, 7H), 6.58 (d, *J* = 8.0 Hz, 12H), 4.82 (d, *J* = 21.8 Hz, 2H), 4.29 (dd, *J* = 36.3, 30.7 Hz, 4H), 4.20 (t, *J* = 6.2 Hz, 2H), 3.59 (d, *J* = 5.3 Hz, 2H), 2.77 (t, *J* = 6.1 Hz, 2H), 2.57 (s, 2H), 2.23 (s, 2H), 0.88 (s, 6H). ^{13}C NMR (151 MHz, $CDCl_3$) δ 191.49, 171.10, 165.74, 165.45, 159.18, 159.16, 146.69, 145.04, 141.52, 141.42, 139.87, 139.77, 139.18, 132.20, 131.86, 128.92, 112.04, 108.69, 108.50, 107.65, 107.53, 107.41, 70.96, 63.64, 62.13, 62.00, 61.98, 60.34, 57.83, 57.74, 49.18, 41.44, 39.71, 33.26, 29.89, 28.14, 20.88, 15.33, 15.30, 14.10.

2.10 Synthesis of compound 8d

Using compound 7b and 3,5-bis[(perfluorophenyl) methoxy] benzoic acid as reactants, according to the reaction procedure of compound 8a, compound 8d (1.67 g, 0.87 mmol) was obtained as a red oily solid with a yield of 75%. MS(MALDI) (M+, $C_{87}H_{51}F_{30}NO_{13}S$): calcd: 1919.26; found: 1920.23. ^{1}H NMR (600 MHz, $CDCl_3$) δ 10.02 (d, *J* = 8.0 Hz, 1H), 7.79 (d, *J* = 16.1 Hz, 1H), 7.27 – 7.18 (m, 5H), 7.18 – 7.15 (m, 4H), 7.09 (d, *J* = 2.1 Hz, 1H), 6.89 (d, *J* = 7.9 Hz, 7H), 6.58 (d, *J* = 8.0 Hz, 12H), 4.82 (d, *J* = 21.8 Hz, 2H), 4.29 (dd, *J* = 36.3, 30.7 Hz, 4H), 4.20 (t, *J* = 6.2 Hz, 2H), 3.59 (d, *J* = 5.3 Hz, 2H), 2.77 (t, *J* = 6.1 Hz, 2H), 2.57 (s, 2H), 2.23 (s, 2H), 0.88 (s, 6H). ^{13}C NMR

(151 MHz, $CDCl_3$) δ 191.49, 171.10, 165.74, 165.45, 159.18, 159.16, 146.69, 145.04, 141.52, 141.42, 139.87, 139.77, 139.18, 132.20, 131.86, 128.92, 112.04, 108.69, 108.50, 107.65, 107.53, 107.41, 70.96, 63.64, 62.13, 62.00, 61.98, 60.34, 57.83, 57.74, 49.18, 41.44, 39.71, 33.26, 29.89, 28.14, 20.88, 15.33, 15.30, 14.10.

2.11 Synthetic chromophore FZL1

compounds 8a (0.79 g, 0.70 mmol) and 2 - (dicyanomethylidene) - 4-methyl-5-phenyl-5 - (trifluoromethyl) - 2,5-dihydrofuran-3-carboxonitrile (0.27 g, 0.84 mmol) were added to a double necked flask filled with argon in anhydrous ethanol (10 mL). After 6 hours of reaction at 65 ℃, concentrated the solution with a rotary evaporator. Flash chromatography of the crude (acetone: hexane = 1:8 to 1:1) over SiO2 obtain a green solid chromophore FZL 1 with a yield of 80% (0.80 g, 0.56 mmol). HRMS (ESI) ([M+ H]+, $C_{91}H_{75}F_3N_4O_7S$): calcd: 1425.5387; found: 1425.5392.^{1}H NMR (600 MHz, $CDCl_3$) δ8.41 – 8.32 (m, 1H, ArH), 8.21 (t, *J* = 8.4 Hz, 3H, ArH), 8.10 – 7.97 (m, 5H, ArH), 7.90 (t, *J* = 7.3 Hz, 1H, ArH), 7.70 (d, 1H, CH), 7.60 – 7.34 (m, 16H, ArH), 7.31 – 7.25 (m, 3H, ArH), 7.24 – 7.14 (m, 6H, ArH), 7.03 – 6.91 (m, 1H, CH), 6.73 (d, *J* = 21.5, 1H, CH), 6.63 (d, 1H, CH), 6.50 (d, 1H, CH), 4.32 – 4.10 (m, 6H, OCH_2), 4.09 – 4.01 (m, 2H, NCH_2), 3.90 (ddt, *J* = 18.4, 10.1, 6.5 Hz, 4H, NCH_2), 3.61 – 3.47 (m, 2H, SCH_2), 3.43 – 3.32 (m, 2H, CH_2), 2.99 – 2.89 (m, 2H, CH_2), 2.82 – 2.72 (m, 4H, CH_2), 2.56 – 2.52 (m, 2H, CH_2), 1.62 (s, 4H, CH_2), 1.01 (d, *J* = 6.5 Hz, 3H, CH_3), 0.93 (d, *J* = 8.6 Hz, 3H, CH_3). ^{13}C NMR (151 MHz, $CDCl_3$) δ175.52, 172.39, 171.20, 162.93, 156.96, 148.61, 147.17, 139.39, 139.34, 136.21, 136.12, 133.74, 132.96, 132.21, 132.06, 131.56, 131.39, 130.31, 129.81, 129.72, 129.69, 129.54, 129.48, 129.43, 129.39, 129.35, 128.42, 128.06, 127.95, 126.73, 126.52, 126.46, 126.38, 126.27, 126.02, 125.34, 125.17, 124.97, 124.80, 123.80, 122.91, 117.44, 112.07, 111.08, 110.60, 62.93, 60.43, 58.52, 46.25, 41.13, 35.13, 34.03, 32.21, 32.09, 31.96, 30.79, 30.42, 28.57, 27.89, 26.94, 23.67, 23.16, 22.76, 21.08.

2.12 Synthetic compound FZL2

Compound 8b (0.72 g, 0.81 mmol) and 2 - (dicyanomethylidene) - 4-methyl-5-phenyl-5 - (trifluoromethyl) - 2,5-dihydrofuran-3-carboxonitrile were placed in flask as raw materials, chromophore FZL2 (0.70 g, 0.59 mmol) was prepared by the procedure of chromophore FZL1, and the green solid product was obtained with 73%. HRMS (ESI) ([M+ H]+, $C_{61}H_{55}F_3N_7O_{13}S$): calcd: 1182.3531; found: 1182.3539.^{1}H NMR (600 MHz, $CDCl_3$) δ 7.98 (d, *J* = 16.0 Hz, 2H, CH), 7.59 – 7.52 (m, 5H, ArH, CH), 7.48 – 7.41 (m, 3H, CH), 6.99 (d, *J* = 16.0 Hz, 1H, CH), 6.78 (d, *J* = 9.0 Hz, 2H, ArH), 6.71 (d, *J* = 7.9 Hz, 6H, CH), 6.56 (d, *J* = 14.6 Hz, 1H, CH), 4.28 (t, *J* = 6.2 Hz, 4H), 4.16 – 4.10 (m, 2H, NCH2), 3.83 (t, *J* = 7.0 Hz, 4H, NCH2), 3.77 (t, *J* = 7.1 Hz, 2H, NCH2), 3.70 (t, *J* = 6.2 Hz, 4H, OCH2), 2.80 (t, *J* = 6.5 Hz, 2H, OCH2), 2.65 (t, *J* = 7.0 Hz, 4H, OCH2), 2.60 (t, *J* = 6.6 Hz, 2H, SCH2), 2.06 (s, 2H, CH2), 1.66 (s, 2H, SCH2), 1.01 (s, 3H, CH3), 0.91 (s, 3H, CH3). ^{13}C NMR (151 MHz, $CDCl_3$) δ 175.49, 170.64, 170.34, 163.01, 156.88, 153.76, 148.60, 147.21, 137.31, 134.24, 131.42, 130.06, 129.56, 128.40, 126.82, 125.73, 125.22, 117.55, 112.23, 111.25, 111.06, 110.60, 77.26, 77.05, 76.84, 63.02, 61.54, 58.54, 49.44, 41.76, 41.09, 34.00, 33.53, 32.87, 30.39, 29.71, 28.59, 27.84.

2.13 Synthetic compound FZL3

Using compound 8c (1.19 g, 0.86 mmol) and trifluoride acceptor as raw materials, chromophore FZL3 (1.08 g, 0.64mmol) was prepared by the procedure of chromophore FZL1, and the green solid product was obtained with 75%. HRMS (ESI) ([M+ H]+, $C_{103}H_{88}F_3N_4O_{13}S$): calcd: 1677.6021; found: 1675.6022. ^{1}H NMR (600 MHz, $CDCl_3$) δ 8.00 (dd, *J* = 26.6, 14.6 Hz, 2H, ArH), 7.55 (dd, *J* = 15.5, 7.5 Hz, 5H, ArH), 7.47 – 7.30 (m, 33H, ArH), 7.26 – 7.20 (m, 6H, ArH), 6.88 (d, *J* = 15.9 Hz, 1H, ArH), 6.84 – 6.82 (m, 1H, ArH), 6.78 (d, 2H, CH), 6.76 (d, *J* = 8.6 Hz, 2H, CH), 6.51 (d, *J* = 14.5 Hz, 1H, CH), 5.00 (d, *J* = 12.4 Hz, 12H, OCH_2), 4.49 (s, 4H, OCH_2), 4.34 (s, 2H, OCH_2), 3.79 (s, 4H, NCH_2), 2.93 (d, 2H, SCH_2), 1.64 (s, 4H, CH_2), 0.99 (s, 3H, CH_3), 0.90 (s, 3H, CH_3). ^{13}C NMR (151 MHz, $CDCl_3$) δ175.51, 171.20, 166.15, 165.83, 159.82, 157.07, 148.79, 147.28, 137.32, 136.38, 131.71, 131.50, 131.40, 129.90, 129.81, 129.73, 129.49, 128.65, 128.63, 128.37, 128.18, 128.15, 127.62, 127.56, 127.54, 126.80, 125.69, 125.15, 117.41, 112.28, 111.10, 108.61, 108.38, 107.57, 70.25, 63.36, 61.96, 60.43, 49.49, 41.68, 41.09, 34.14, 30.38, 29.72, 28.62, 27.85, 21.07.

2.14 Synthetic compound FZL4

Using compound 8d (1.67 g, 0.87 mmol) and trifluoride acceptor as raw materials, chromophore FZL4 (1.50 g, 0.68mmol) was prepared by the procedure of chromophore FZL1, and the green solid product was obtained with 78%. HRMS (ESI) ([M+ H]+, $C_{103}H_{58}F_{33}N_4O_{13}S$): calcd: 2217.3194; found: 2317.3190. ^{1}H NMR (600 MHz, $CDCl_3$) δ 8.02 (s, 1H, ArH), 7.93 (d, *J* = 15.9 Hz, 1H, ArH), 7.54 (ddd, *J* = 17.4, 15.5, 7.6 Hz, 5H, ArH), 7.44 (d, *J* = 12.3 Hz, 1H, ArH), 7.37 (d, *J* = 8.8 Hz, 2H, ArH), 7.28 (s, 1H, ArH), 7.27 (d, *J* = 2.3 Hz, 2H, ArH), 7.25 (d, *J* = 2.3 Hz, 3H, CH), 6.86 (t, *J* = 17.2 Hz, 1H, CH), 6.82 – 6.69 (m, 5H, ArH), 6.51 (d, *J* = 14.6 Hz, 1H, CH), 5.06 (d, *J* = 18.6 Hz, 12H, OCH_2), 4.54 (t, *J* = 5.9 Hz, 4H, OCH_2), 4.36 (t, *J* = 6.2 Hz, 2H, OCH_2), 3.88 (t, *J* = 5.8 Hz, 4H, NCH_2), 2.94 (t, *J* = 6.4 Hz, 2H, SCH_2), 1.64 (d, *J* = 11.5 Hz, 4H, CH_2), 0.99 (s, 3H, CH_3), 0.90 (s, 3H, CH_3). ^{13}C NMR (151 MHz, $CDCl_3$) δ175.49, 171.19, 165.78, 165.44, 163.00, 159.19, 159.17, 153.86, 146.70, 145.08, 145.06, 141.56, 141.46, 139.91, 139.81, 139.25, 132.04, 131.77, 131.40, 129.89, 129.71, 129.67, 129.52, 128.38, 126.78, 125.65, 125.02, 123.00, 121.10, 117.43, 112.18, 110.57, 108.84, 108.60, 107.69, 107.50, 107.40, 70.98, 62.09, 60.41, 57.80, 49.30, 41.60, 41.01, 33.99, 30.29, 29.71, 28.53, 27.74, 21.05.

3. NMR pictures

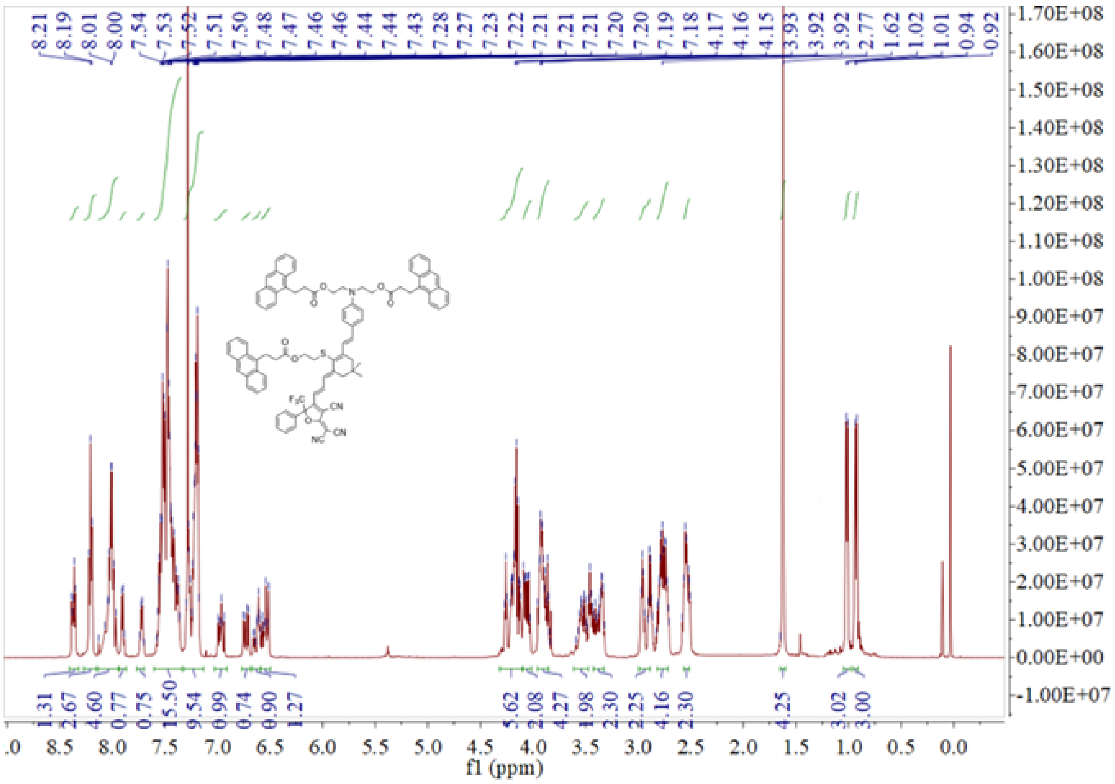

Figure S1. ^{1}H-NMR spectrum of Chromophore FZL1

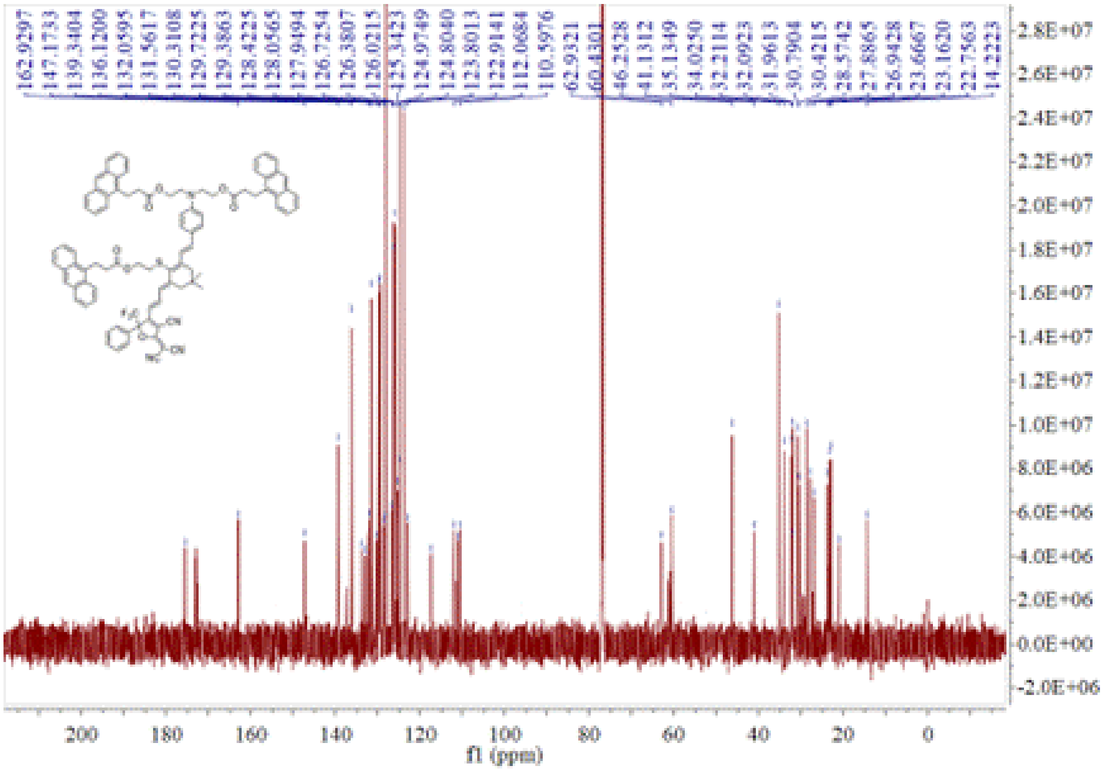

Figure S2. ^{13}C-NMR spectrum of Chromophore FZL1.

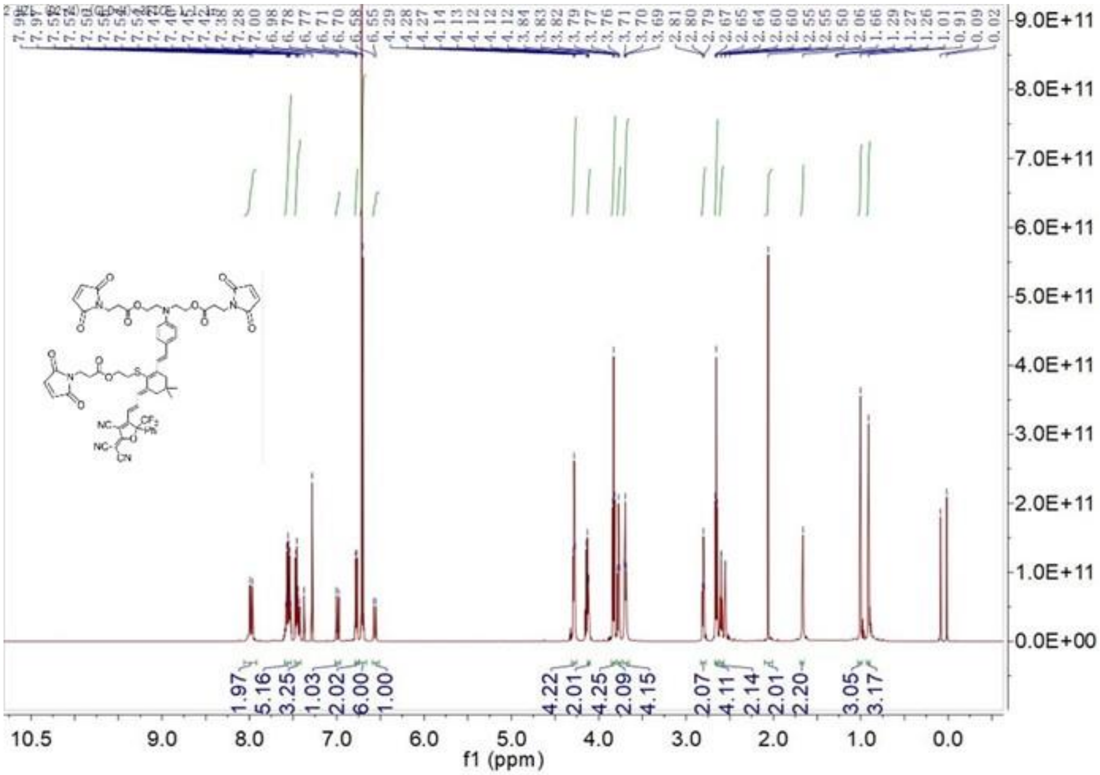

Figure S3. ^{1}H-NMR spectrum of Chromophore FZL2.

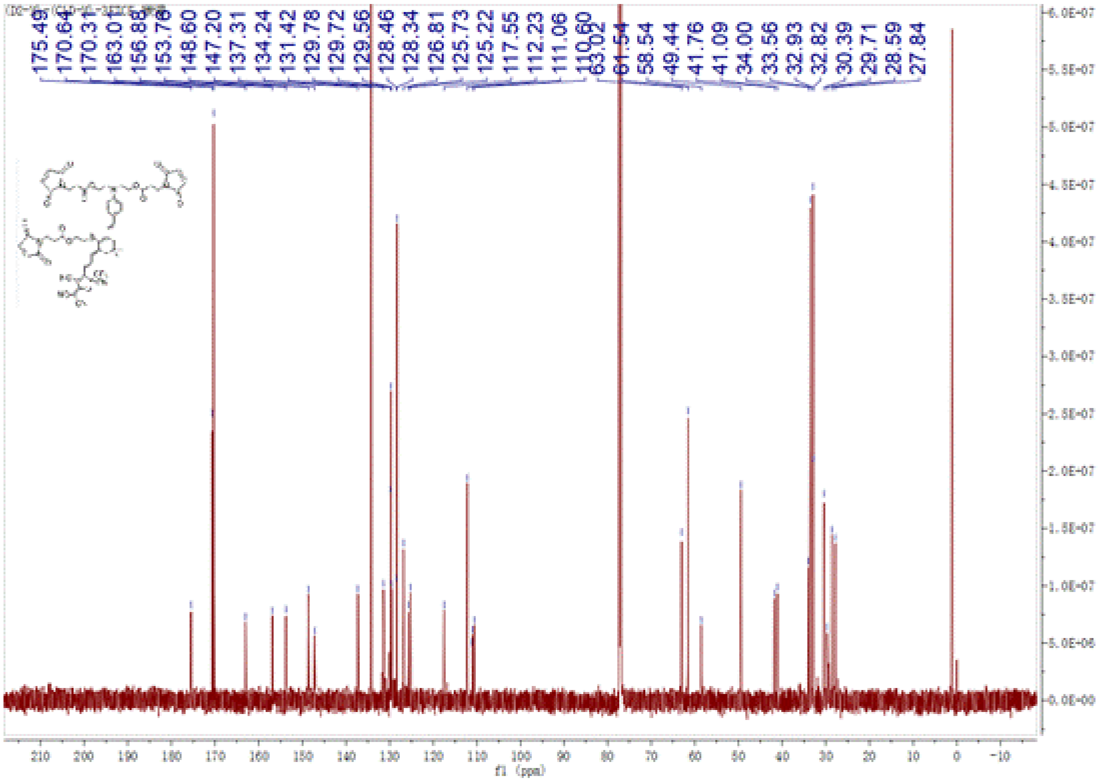

Figure S4. ^{13}C-NMR spectrum of Chromophore FZL2.

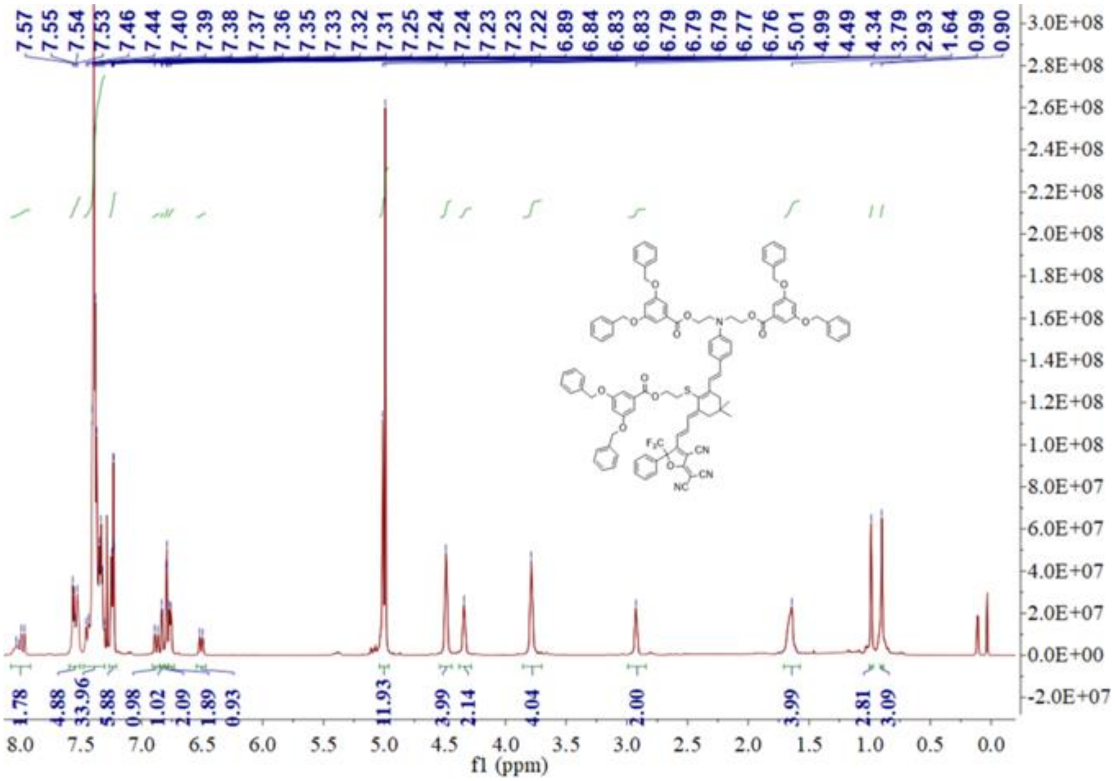

Figure S5. ^{1}H-NMR spectrum of Chromophore FZL3.

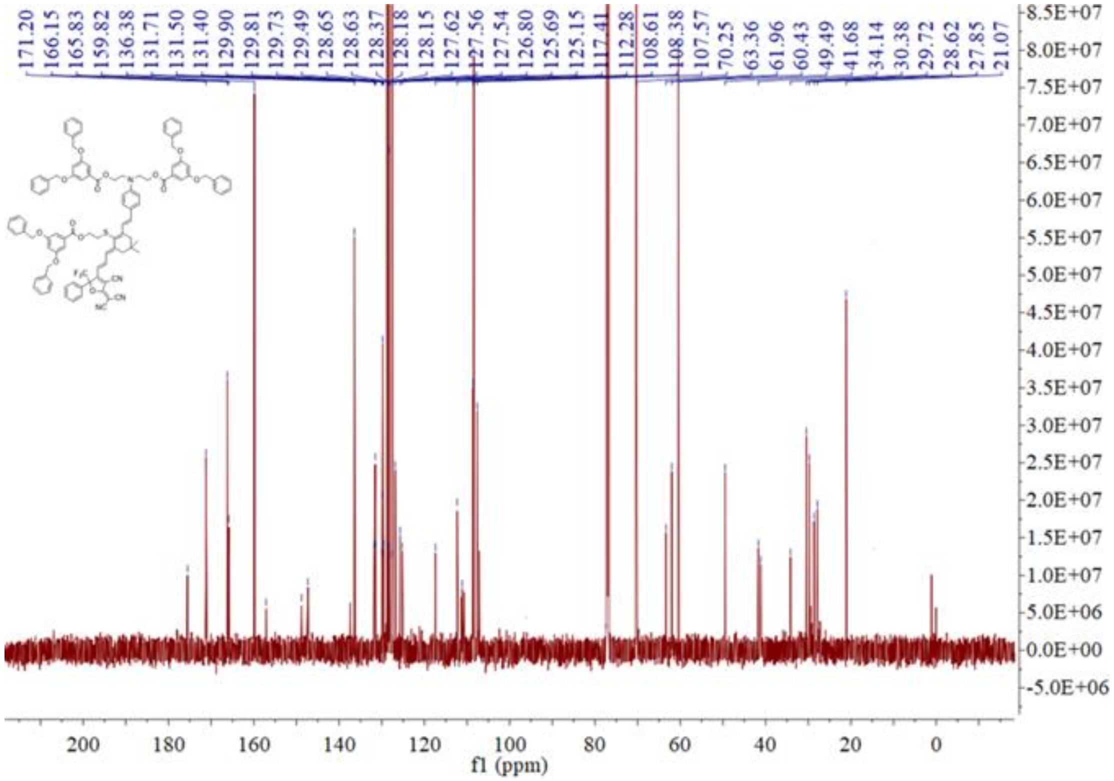

Figure S6. ^{13}C-NMR spectrum of Chromophore FZL3.

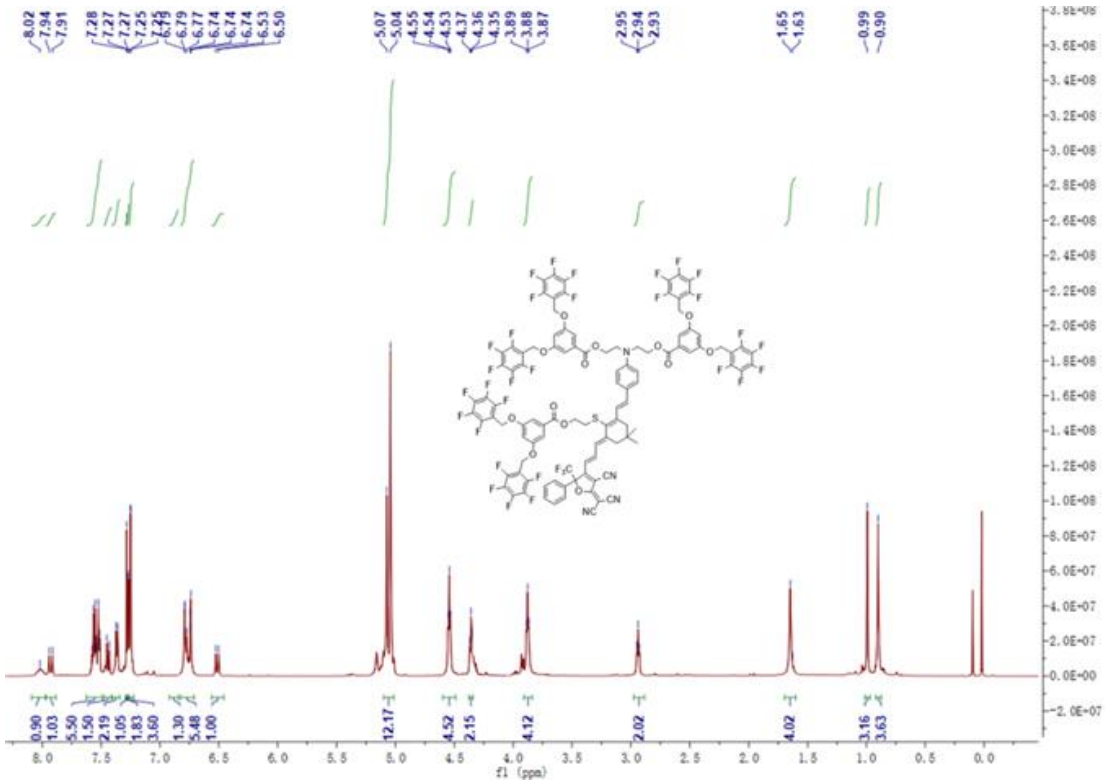

Figure S7. ^{1}H-NMR spectrum of Chromophore FZL4.

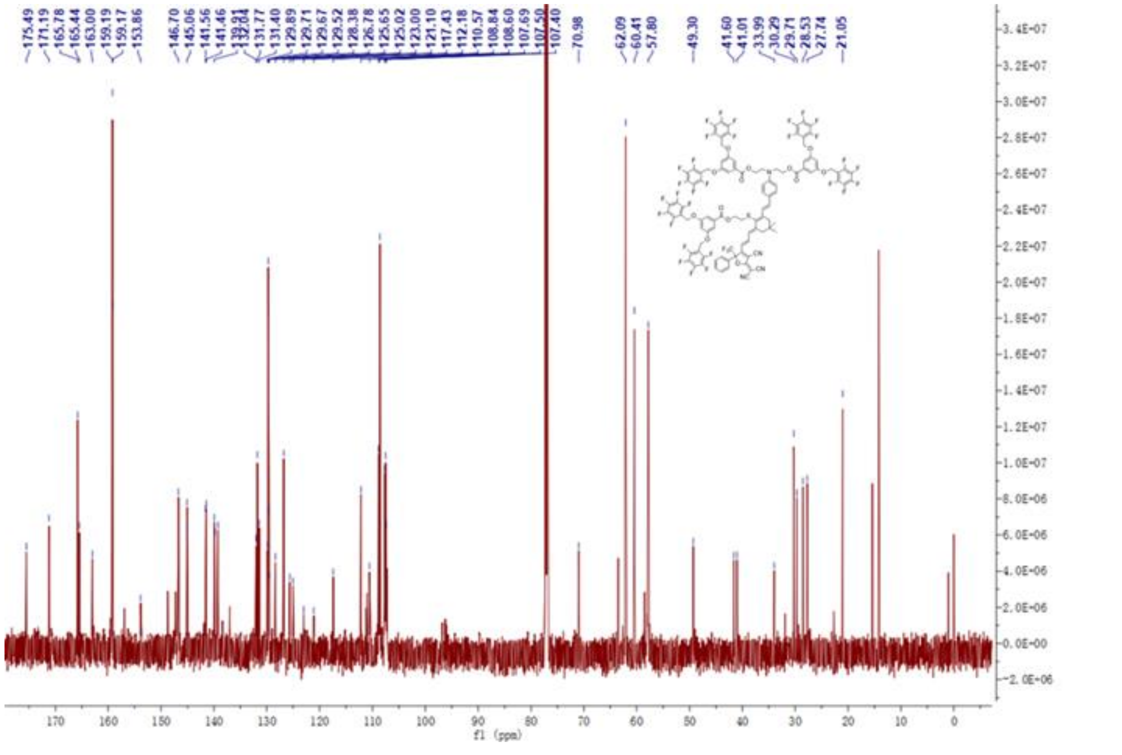

Figure S8. ^{13}C-NMR spectrum of Chromophore FZL4.

4. UV-Vis Absorption Spectroscopy

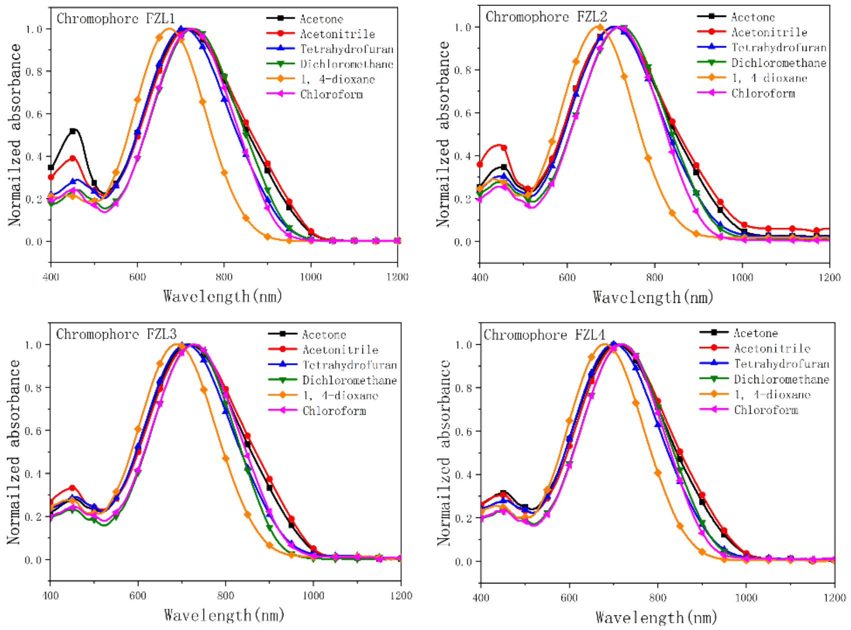

Figure S9. Normalized UV-Vis absorption spectra of chromophores FZL1-4 in seven aprotic solvents

5. Differential Scanning Calorimetry testing

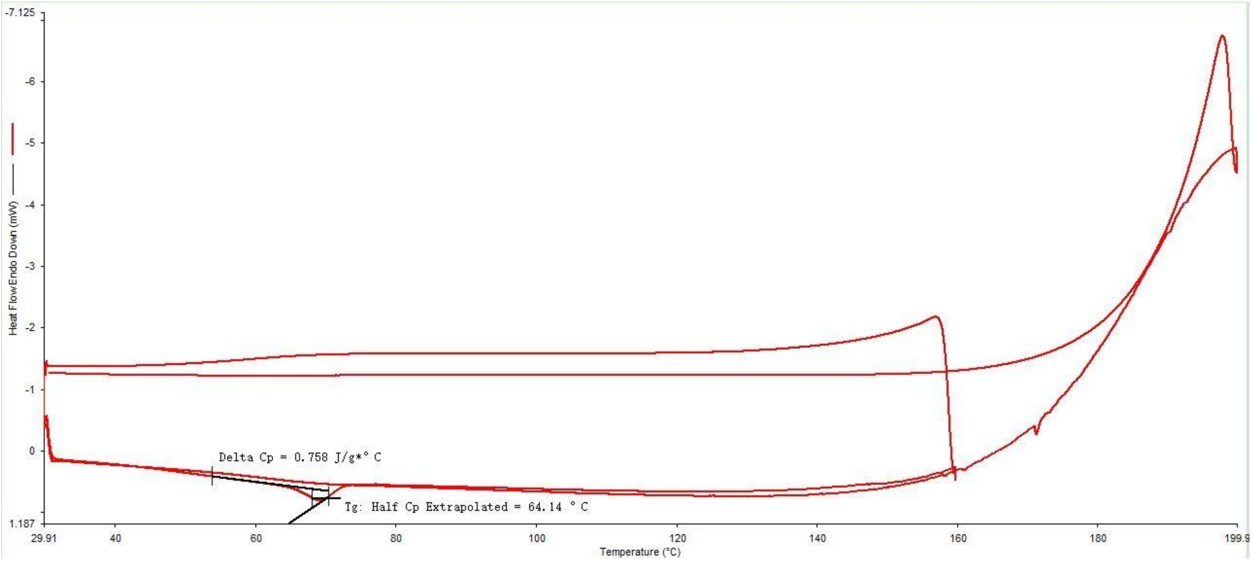

Figure S10. DSC curves for crosslinking chromophore FZL1

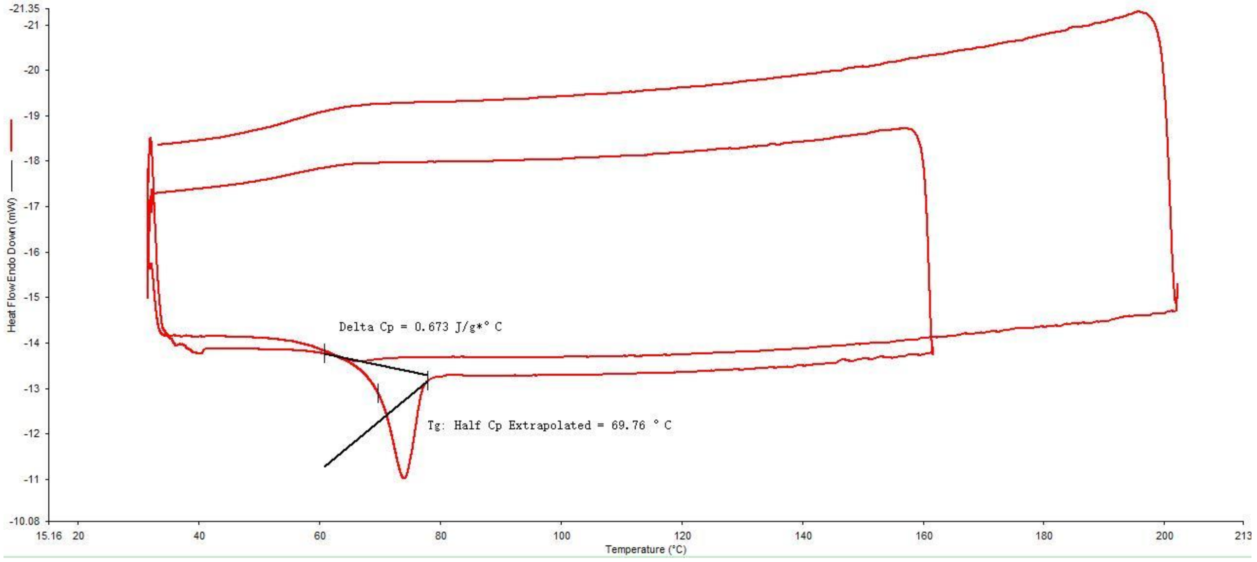

Figure S11. DSC curves for crosslinking chromophore FZL2

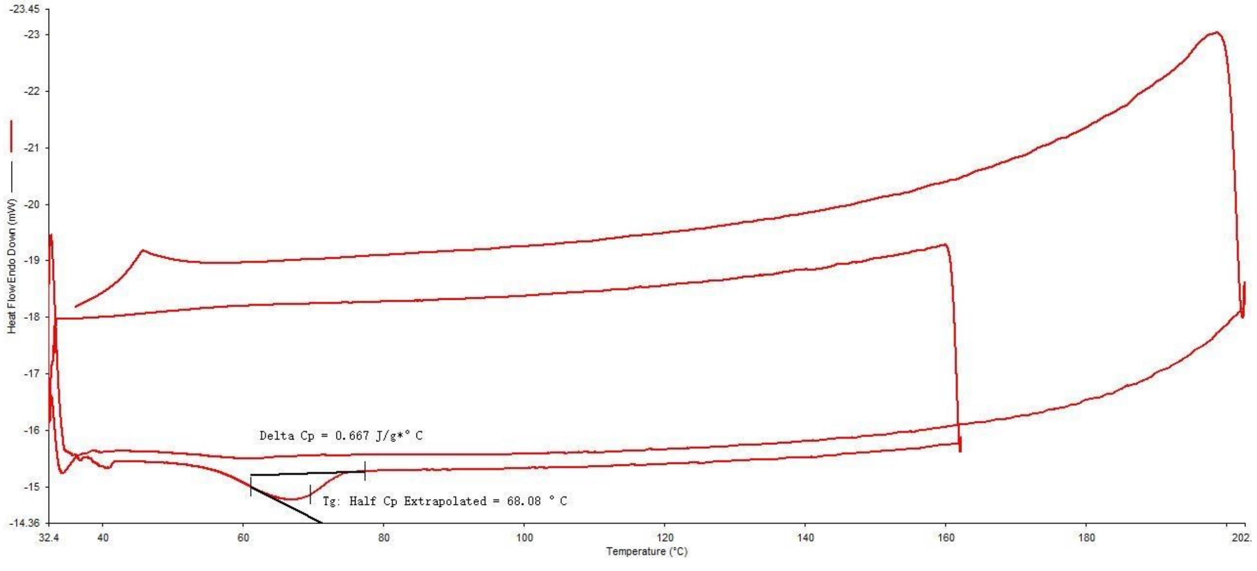

Figure S12. DSC curves for crosslinking chromophore FZL3

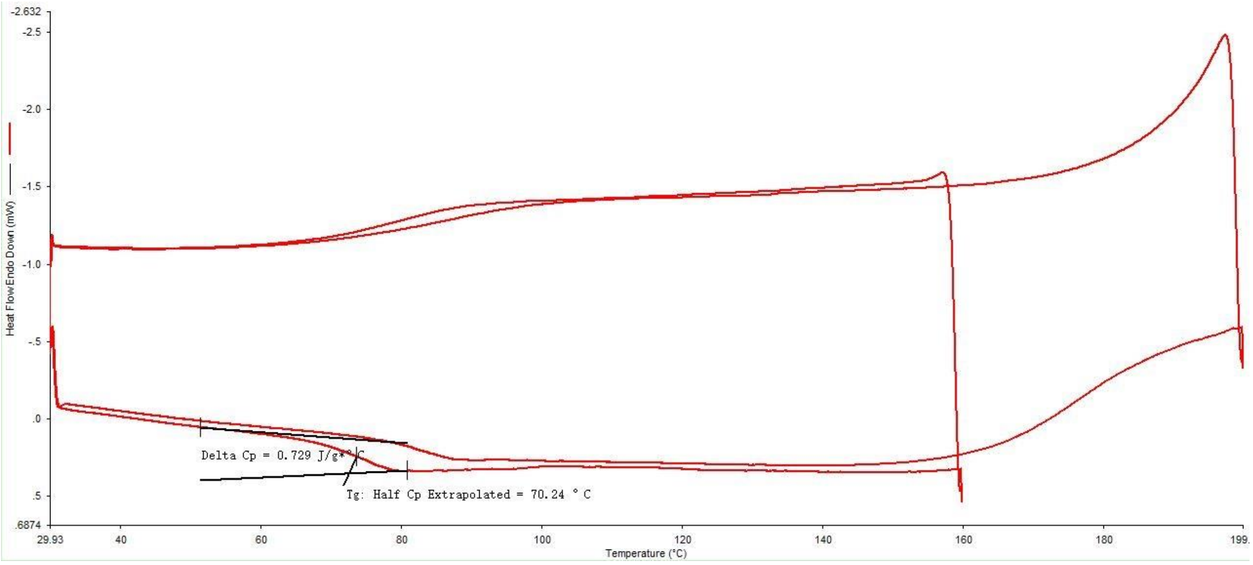

Figure S13. DSC curves for crosslinking chromophore FZL4

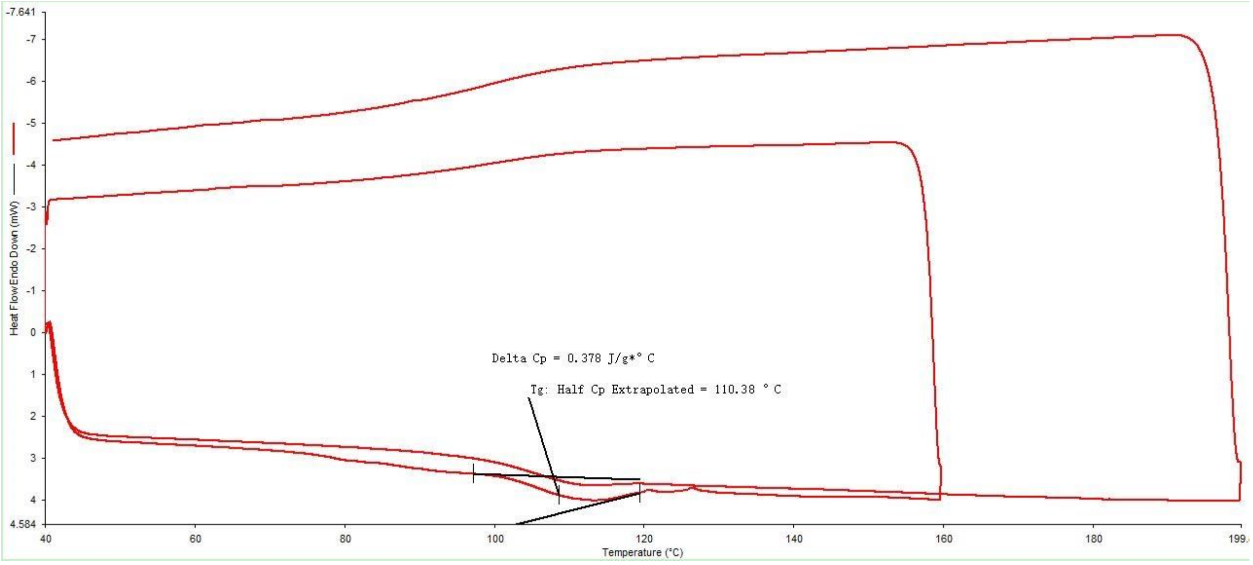

Figure S14. DSC curves for crosslinking chromophores 1：1 FZL1/FZL2 before crosslinking

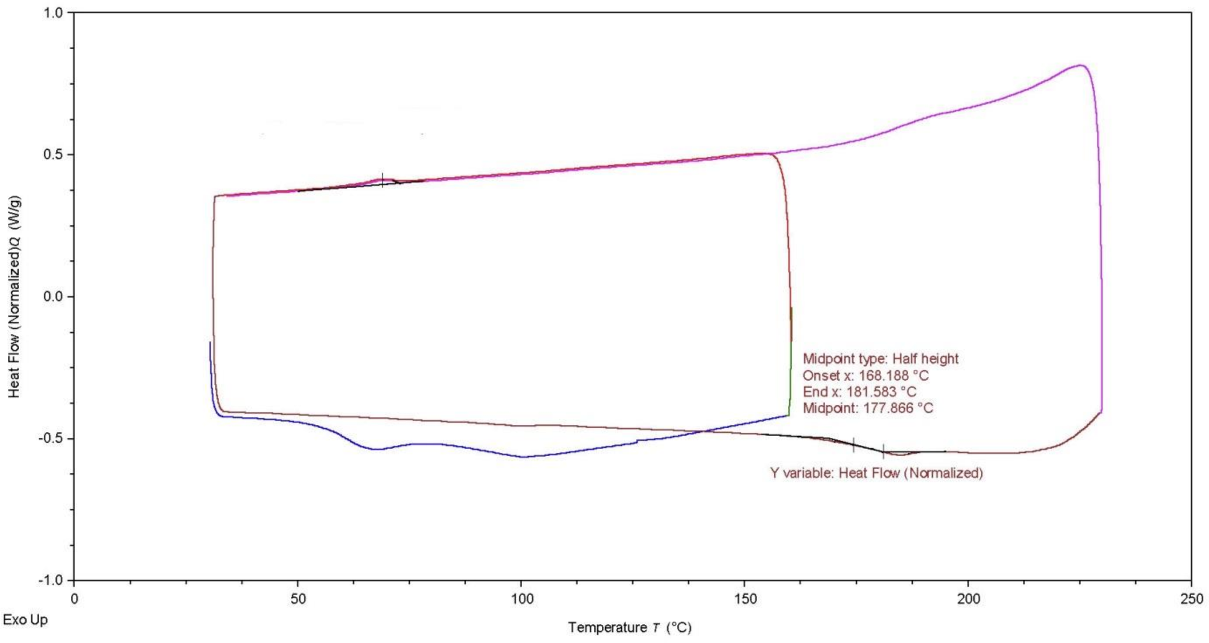

Figure S15. DSC curves for crosslinking chromophores 1：1 FZL1/FZL2 after crosslinking

6. DFT Calculations

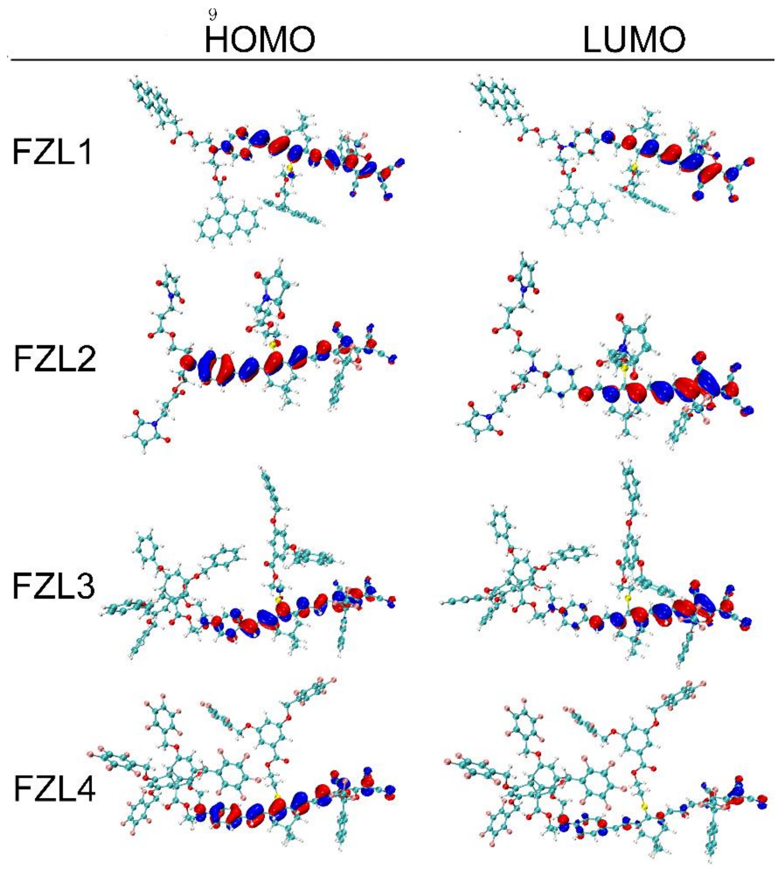

Figure S16. HOMO-LUMO energy level orbital diagram of chromophores FZL1-FZL4

7. Properties of the state-of-the-art organic EO materials

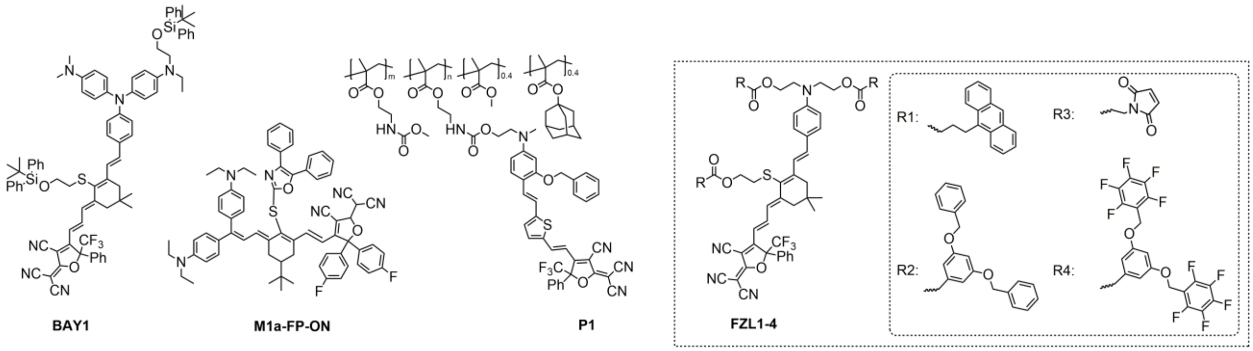

Figure S17. Chemical structures of the state-of-the-art organic EO materials.

Table S1 Properties of the state-of-the-art organic EO materials[1-3]

Cmpd	T_d (°C)	T_g (°C)	β_{tot}^a (10^{-30}esu)	max. r_{33}/(pm/V)
**BAY1**	205	84	2941	460 or 1100 （with TiO_2）
**M1a-FP-ON**	233	--	376	127
**P1**	--	172	--	223
**FZL1-4**	274-307	64-178	781-876	266-308

[a] was the first-order hyperpolarizability in vacuum calculated from DFT calculations.

Supplementary Table S1 summarizes the properties of the reported state-of-the-art organic EO materials, including monolithic chromophore (BAY1), guest-host systems (M1a-FP-ON) and polymer (P1). Although the each single performance of FZL series materials was not the highest among them, it is comparable to state-of-the-art values of the previously reported organic EO materials. Meanwhile, it possesses the highest thermal decomposition temperature (T_d) and glass transition temperature (T_g) among the 4 materials shown in Suppl. Table S1, indicating excellent high-temperature thermal stability. Hence, the presented FZL series materials stands out in terms of the high-temperature stability and EO efficiency in a well-balanced manner.

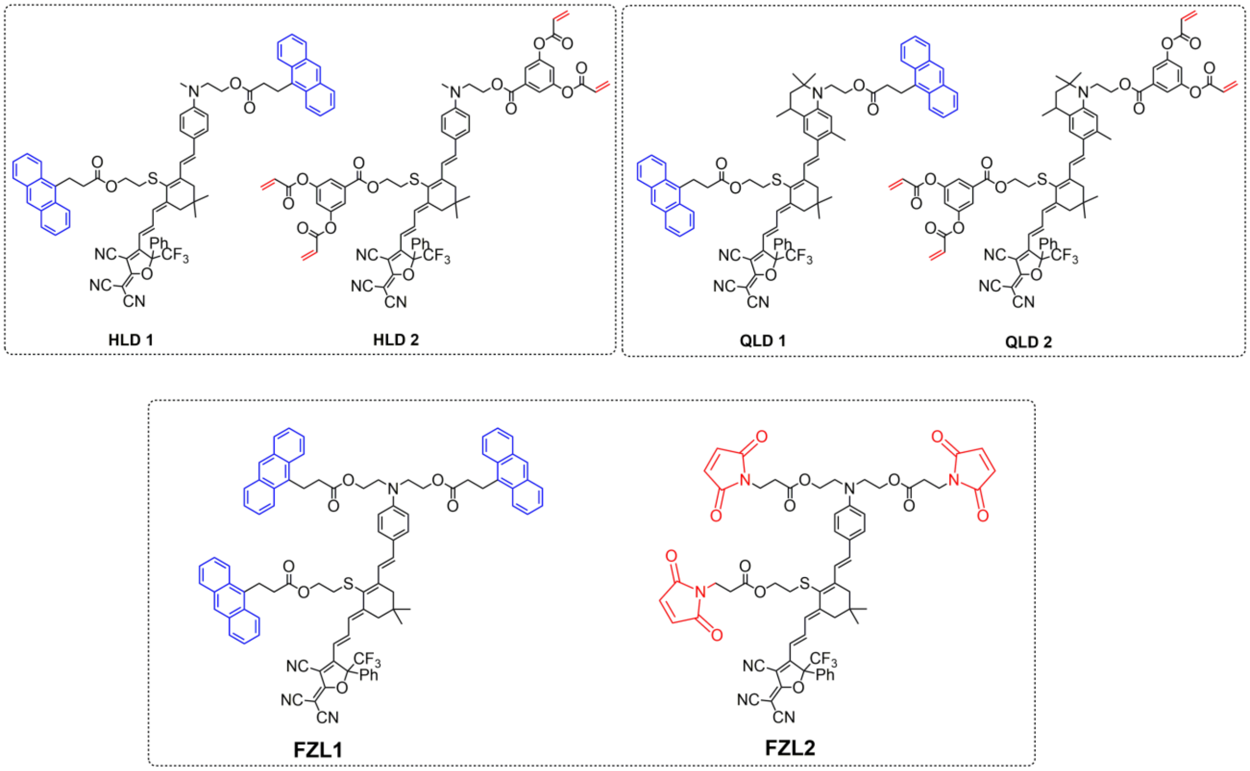

Figure S18. Chemical structures of the binary cross-linked Chromophore HLD1-2, QLD1-2 and FZL1-2.

8. Comparison of different crosslinking systems

Table S2 Properties of the state-of-the-art organic EO materials

Cmpd	T_d (°C)	T_g (°C)	Poling time and temperature	max. r_{33}/(pm/V)
**2:1 HLD1/HLD2**	231/317	174	160 °C 60min	290
**2:1 QLD1/QLD2**	273/317	185	160 °C 60min	327
**1:1 FZL1/FZL2**	274/298	178	135 °C 30min	266

Supplementary Table S2 summarizes the properties of the reported state-of-the-art organic binary pure chromophore cross-linking material HLD1-2, QLD1-2 and FZL1-2. Compared to the reported advanced binary cross-linked electro-optic materials, the performance (T_g and electro-optic coefficient) of FZL1-2 is comparable to HLD1-2 and QLD1-2. Similar conjugated structures result in similar first-order hyperpolarizabilities and electro-optical coefficients. And the three functionalized groups are also more conducive to crosslinking. The cross-linking reaction of anthracene-maleimide occurs faster than that of anthracene-acrylate, so the poling temperature of FZL1-2 was lower and the time was faster, which is more conducive to the stability of chromophore and energy saving.

9. Electric field induced polarization .

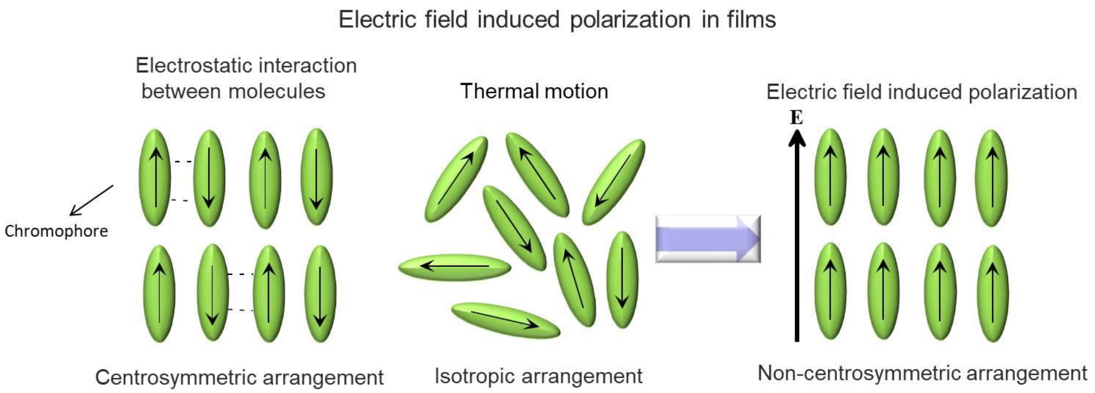

Figure S19. Three-dimensional alignments of chromophores in the film states.

Chromophore molecules usually have a large dipole moment, so in the film, Chromophore molecules will appear antiparallel stacking arrangement（Centrosymmetric arrangement）. At this point, the molecules cannot exhibit electro-optical activity. When we heat the electro-optic film, the thermal motion of molecules becomes intense, and the molecules gradually appear isotropic arrangement in the film. When the temperature reaches near the glass transition temperature of the thin film, under the action of an electric field, dipole molecules will undergo a turning, some molecules exhibit Non-centrosymmetric arrangement and exhibit nonlinear optical activity.

10. Long-term alignment stability test

The Long-term and/or high-temperature alignment stability test was conducted follow the steps below：We selected three electro-optic films 1:1 FZL1/FZL2 that achieved the maximum electro-optic coefficient when the poling condition (poling voltage, temperature and time) was carefully explored to achieve the optimization. This also applies to films 1:1 FZL1/FZL4 and1:1 FZL3/FZL4. These films were placed in a vacuum drying oven, heated to the desired temperature, and placed for 500 hours. The electro-optical coefficients of these thin films were measured again and compare them with the previous values.The three poled electro-optic films 1:1 FZL1/FZL2 could still maintain more than 99.43%、99.87% and 99.89%, respectively, of the original electro-optic coefficient. The average value was 99.73%±0.30%. The three poled electro-optic films 1:1 FZL1/FZL4 could still maintain more than 97.21%、95.56% and 98.56%, respectively, of the original electro-optic coefficient. The average value was 97.11%±1.45%. The three poled electro-optic films 1:1 FZL3/FZL4 could still maintain more than 97.69%、 98.72% and 98.28%, respectively, of the original electro-optic coefficient. The average value was 98.23%±0.54%.

11. Reference

1. G.-W. Lu, J. Hong, F. Qiu, A. M. Spring, T. Kashino, J. Oshima, M.-a. Ozawa, H. Nawata and S. Yokoyama, *Nature Communications*, 2020, **11**, 4224.
2. D. Zhang, J. Zou, W. Chen, S.-M. Yiu, M.-K. Tse, J. Luo and A. K. Y. Jen, *Chemistry of Materials*,

2022, **34**, 3683-3693.

3. H. Xu, D. L. Elder, L. E. Johnson, Y. de Coene, S. R. Hammond, W. Vander Ghinst, K. Clays, L. R. Dalton and B. H. Robinson, *Advanced Materials*, 2021, **33**, 2104174.
